# A high-resolution, multi-method palaeoenvironmental record from pan sediments at Maralaleng Pan, southern Kalahari basin

**DOI:** 10.1371/journal.pone.0357461

**Published:** 2026-09-17

**Authors:** Inèz Faul, Taylor Grandfield, Chris Green, Elena A. Hensel-Karamalakidou, Phillip Segadika, Rowena Winterhalder, Sarah Mothulatshipi, Stefan Dreibrodt, Nils Andersen, Marine Frouin, Michaela Ecker

**Affiliations:** 1 Institute of Prehistoric and Protohistoric Archaeology, Kiel University, Kiel, Germany; 2 Department of Geosciences, Stony Brook University, United States of America; 3 Institute for Ecosystem Research, Kiel University, Kiel, Germany; 4 Department of National Museum and Monuments, Gaborone, Botswana; 5 Department of History, University of Botswana, Gaborone, Botswana; 6 State Office for Monument Preservation Baden-Württemberg, Germany; 7 Department of Geosciences, University of Tübingen, Germany; 8 Leibniz Laboratory for Radiometric Dating and Stable Isotope Research, Kiel University, Kiel, Germany; 9 Archaeology Department, Kimberley, South Africa; Hefei University of Technology School of Resources and Environmental Engineering, CHINA

## Abstract

Human traces in semi-arid environments such as the Kalahari have been linked to the presence of water in the landscape. However, low organic preservation and an uneven distribution of studied sites have resulted in a gap in the knowledge of the environmental conditions under which *Homo sapiens* adapted to drylands in the Late Pleistocene. The Kalahari boasts numerous seasonal waterbodies (pans) that may provide stratified sequences containing a variety of palaeoenvironmental proxies. Despite this, these environmental archives have rarely been used. In this study, we report on a multi-method investigation of the palaeoenvironmental context of Maralaleng Pan, a pan site with archaeological material in the southern Kalahari basin. Geoarchaeological methodologies, luminescence dating, and plant wax biomarkers have been combined to create a high-resolution palaeoenvironmental record. This contribution reveals that pan sediments from Maralaleng Pan were deposited in an environment characterised by alternating processes indicative of wet (lake) and dry (evaporative) conditions. The integration of our results with the archaeological investigation of Maralaleng Pan suggests that hominins frequented the landscape during a fluctuating lacustrine environment in MIS 5, while the greatest moisture availability occurred during MIS 3.

## 1. Introduction

The Middle Stone Age (MSA) in Africa starts at ~300 ka, and coincides with the emergence of anatomically modern humans [1–4]. Sites with early MSA lithic typologies in southern Africa date to between 280 ka and 130 ka [4,5]. The MSA corresponds with several evolutionary milestones, such as blade technology [6], cooked starches associated with hearths [7], engraved ochre [8], and shellfish exploitation on the coast [9]. Glacial and interglacial cycles are split into Marine Isotope Stages (MIS) and were major drivers of environmental and landscape change during the MSA (MIS 8−2) [2,10]. MIS 5 and MIS 3−2 have been of special interest for archaeological and palaeoenvironmental research. MIS 5 includes the last interglacial before the present [2,11,12] and corresponds with the transition from wide-spread early MSA lithic technologies to regional variations in stone tool technology. MIS 3−2 includes the Last Glacial Maximum (LGM) (MIS 2), the end of the MSA, and the start of the Later Stone Age (LSA). The exact start of the LSA and end of the MSA is disputed, as the transition varies throughout different regions (e.g., discussed in [13]).

The Kalahari is landlocked in the interior of southern Africa and past environmental changes impacted the local semi-arid landscape in different ways compared to the well-researched southern Cape coast [14,15]. Palaeoenvironmental information from the southern African drylands is vague and integrating these results with each other has been difficult due to a lack of near-continuous terrestrial records [e.g., 16]. Despite this uneven record, palaeoenvironmental research from the southern Kalahari basin has shown that this semi-desert is a dynamic environment that played an important role in hominin migration, site choices, and adaptation [17–24]. Previous contributions [25] suggest that human presence in the Kalahari basin can be linked to seasonal waterbodies known as pans. Dry lakebeds and pan floors in the Kalahari were used as knapping sites during the Late Pleistocene [20,23,26]. Therefore, linking changing pan environments with archaeological material is vital for understanding how humans adapted to the southern Kalahari landscape.

**Fig 1:**
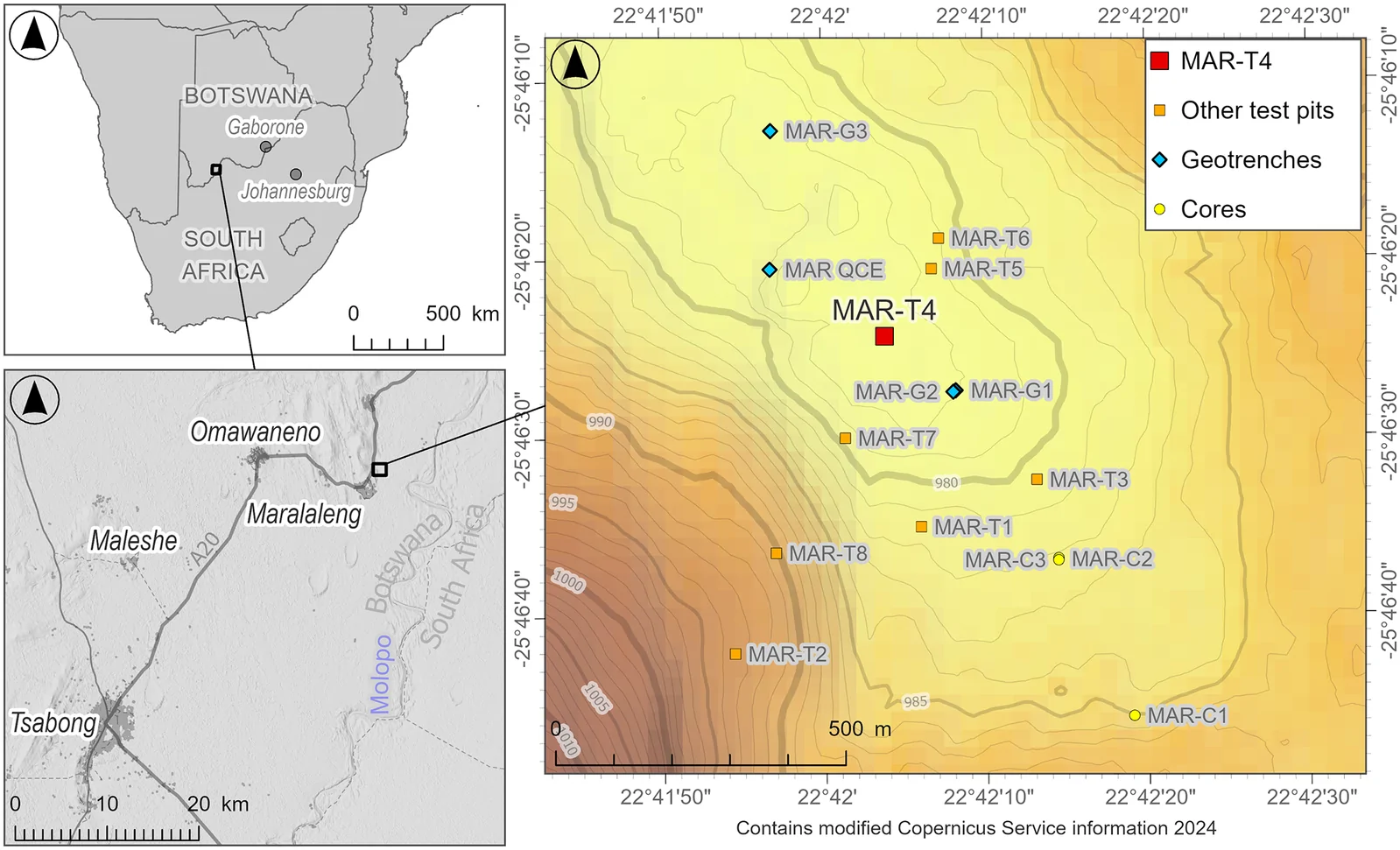
The location of the Kgalagadi district within southern Africa (upper left); and the location of MAR Pan within the Kgalagadi district in south-western Botswana (bottom left). The sampling contexts of all MAR Pan samples analysed to date are labelled with blue diamonds (geotrenches), yellow circles (cores), and orange squares (test pits) (MAR-T1, MAR-T2; MAR QCE 1; MAR-G1 can be found in [25]). The main test excavation analysed in this contribution (MAR-T4) is labelled with a red square. This image contains modified Copernicus Service Information 2024.

Maralaleng Pan (MAR Pan) is an archaeological site located near Maralaleng Village in the Kgalagadi district in south-western Botswana (Fig 1), which has yielded Pleistocene stone tool assemblages [25,27]. This study investigates palaeoenvironmental conditions at MAR using a multi-method approach, which includes Energy Dispersive X-ray fluorescence (ED-XRF), X-ray diffraction (XRD), particle size by laser diffraction, magnetic susceptibility, micromorphology, Optically Stimulated Luminescence (OSL) dating, carbonate stable isotopes and plant wax biomarkers (Fig 1). The new palaeoenvironmental results and OSL dates from MAR are then considered alongside comparable data from the wider Kalahari region to provide a broader perspective on the conditions encountered by hominins as they navigated this landscape.

## 2. Regional setting

### 2.1. The southern Kalahari Basin

In this contribution, the ’Kalahari’ refers to the cratonic basin located in southern Africa, which is covered by Kalahari Group sediments, the most prominent of which is the layer of red Kalahari sand that covers most of the landscape [28]. Bush and shrub savannah account for the sparse vegetation cover throughout the southern Kalahari basin [29]. Our research area falls within the Savannah biome [30]. The southern Kalahari is host to thousands of round depressions known as pans, which become partly saturated during the rainy season in the austral summer months [28,31,32].

The southwest of Botswana is the country’s most arid region (150–300 mm rainfall per annum [33], concentrated during the summer months). The main driver of regional climate dynamics is the convergence of the Intertropical Convergence Zone (ITCZ), which brings summer rainfall inland from the Indian Ocean and cold fronts from the Atlantic (westerlies) in the winter. Rainfall within the summer rainfall zone is, however, highly variable [34]. The summer rainfall zone has been divided into three sections, namely the western part (which has an annual mean precipitation of 564 mm), the eastern part (which has an annual mean precipitation of 706 mm), and the mixed zone (which has an annual mean precipitation of 469 mm) [34]. South-western Botswana is located in the mixed summer rainfall zone. The most common type of dunes in the Tsabong area are vegetated lunette dunes, which often occur on the (southern) leeward side of the pans. Near-surface moisture fluctuations in the sediment result in the formation of duricrusts, such as calcretes, silcretes, and intergrade duricrusts. Multiple authors have analysed Kalahari duricrusts in the last decades [35–42], but very few have studied these sediments at Pleistocene archaeological sites [25,27]. The southern Kalahari basin landscape was characterised by different climatic and environmental conditions during the Pleistocene than the current prevailing conditions [19–21,43].

### 2.2. Maralaleng Pan

MAR is located in the Kgalagadi district of Botswana, ≈ 40 km east of the administrative capital, Tsabong [25,27]. MAR is a vegetated pan with an area of approximately 1.55 km^2^. Despite rainfall being concentrated in the summer months, the evaporation rate is high enough to create a surface moisture deficit, which causes most pans to contain water for only days or weeks [32]. The most prominent features are the duricrust formations in the pan, which are visible in a former gravel extraction pit on the northern side of the pan. Lithics made of local quartzite are scattered across the landscape, but are most abundant on the pan’s sand-covered quartzite outcrops on the north-western to south-eastern slopes. During sheet wash and heavy rainfall events, sediment (and some smaller lithics) is moved downslope, resulting in the redeposition of lithics. During drying periods, deflation processes cause the light sediment particles to be redeposited back onto the ridges [25]. These deflation processes often lead to lunette dune formation on the pan’s leeward side. However, these dunes can also form as a result of sediment deposition following erosion of other nearby dune systems [e.g., 32] and by wave-action [44].

## 3. Materials and methods

### 3.1. Field work and sampling

**Fig 2:**
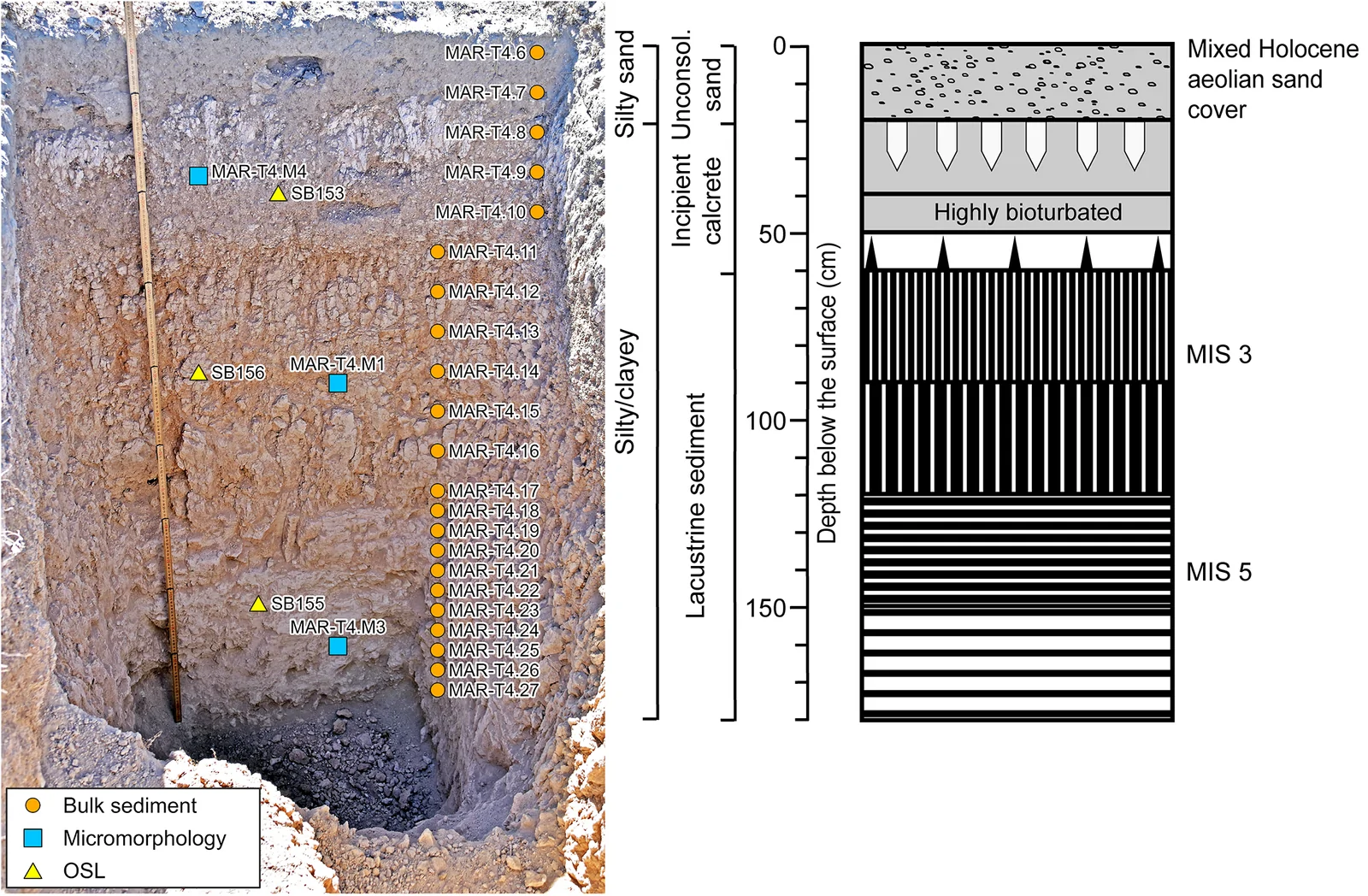
Maralaleng Pan Test Pit 4 (MAR-T4). Left: Sampling locations for bulk sediment, carbonate stable isotopes, OSL, and micromorphology samples in the MAR-T4 profile. Samples for n-alkanes were taken next to the bulk sediment samples on the right-hand side of the profile every 10 cm (Table 4). Right: Field drawing and interpretation of the MAR-T4 Profile.

MAR-T4 is a 1.7 m deep archaeological test pit that was selected due to its proximity to the pan centre, which was expected to yield the most continuous sediment sequence. During the rainy season, this part of the pan can become waterlogged (Fig 1). An archaeological excavation protocol [27] was followed to a depth of 50 cm below the surface, when no more lithics were found. Thereafter, the pit was extended as a geotrench using a pickaxe and shovel. Bulk sediment was sampled every 5 cm from the bottom (169 cm) upwards until 114 cm. Following this, every 10 cm was sampled to the surface (Fig 2). The bottom section of the profile was sampled at a higher resolution than the upper parts, as it was considered to be part of a lake sediment sequence (based on field observations). Three micromorphology samples were taken, namely MAR-T4.M4 (38 cm below the surface), MAR-T4.M1 (90 cm below the surface), and MAR-T4.M3 (156 cm below the surface). The two upper samples were taken using Kubiena boxes as the sediment was too loose to remain intact enough for a Plaster of Paris micromorphology block. The lowermost sample (MAR-T4.M3) was taken by cutting the block out of the profile and stabilising it with Plaster of Paris, as the sediment was cemented.

### 3.2. Bulk sediments

The bulk sediment samples were divided into “columns” samples (referring to duricrusts), and “inter-columns” samples (referring to the ‘modern’ and ‘Holocene’ sands that infiltrated in-between the duricrust columns due to sediment shrinkage and cracking). This separation was done for all samples from 119 to169 cm below surface, as this was where sand infiltration stopped according to field observations. All bulk sediment samples were homogenised into a < 2 mm fraction using a mortar and pestle. The samples used in Total Organic Carbon (TOC), Total Inorganic Carbon (TIC), carbonate stable isotopes, and XRD analyses were further homogenised into a fine powder using a ball mill (Retsch MM400). These powdered samples (excluding the carbonate isotope samples) were dried in an oven at 40°C for a minimum of one week before analysis.

All homogenised ball-mill samples (columns, inter-columns, and unseparated samples) were then analysed for their elemental compositions using a NITON XL2 plus Energy Dispersive XRF device. Approximately 5 grams per sample of homogenised sediment was measured using the “Cu/Zn mining modus”, and each sample was measured for 300 seconds (120 seconds main filter; 120 seconds light filter) [45–47]. A standard sample (TILL3, 31M, O’Brien Mine, near Cobalt, Ontario) was measured regularly to ensure measurement quality and to calibrate the results. Selected samples were also analysed under a Scanning Electron Microscope (SEM) (Hitachi TM4000 Plus equipped with a Bruker Quantax 75 ED-micro-XRF).

Only ‘column’ samples below 119 cm were analysed for their mineral composition using X-ray Diffraction (XRD). A D8 Discover by Bruker AXS with Cu K-α radiation and a wavelength 1.54 Å, step size of 0.037° 2 theta, count time of 2 s/step, and measuring range of 2°-70° 2 theta was used. Minerals from the XRD analysis were identified using the High Score Plus software by PANalytical version 4.8 (4.8.0.255.18) [48].

A Euro EA (Elemental analyser from Hekatech) machine was used for the TIC/TOC analysis. Twenty-two samples from MAR-T4 (all “column” samples) were analysed and treated with 40% phosphoric acid (H_3_PO_4_) during TIC analysis. The TOC levels in dry sediment were determined by subtracting C% from IC (See Supplementary Information 3, Table S8 in S2 File for raw TIC/TOC data). The CaCO_3_ content was determined by multiplying the IC by the standard multiplier, 8.33 [49]. The TOC% content can be used as a proxy for vegetation density, the accumulation of biomass, and fluvial input; while TIC% content in combination with stable carbon and oxygen isotopes can be used as a proxy for evaporation [50] and therefore, duricrust formation.

All homogenised (<2 mm) samples underwent magnetic susceptibility analysis. Magnetic susceptibility measures the extent to which materials can become magnetised. This relies mostly on the presence of Fe-bearing minerals [51]. The lab processing protocol for magnetic susceptibility described in [52] was applied. Each sample was measured in three replications of 10 g each on a Bartington MS2B susceptibility meter. After every third sample, a 1% Fe_3_O_4_ (magnetite) sample was measured. The resolution of this machine is 2*10−6SI, measuring range 1–9999*10–5 SI with a systematic error of 10% [52]. All measurements were obtained on both low (0.465 kHz) and high (4.65 kHz) frequencies. A particle size analysis was carried out on all (<2 mm) MAR-T4 samples, using a Mastersizer 2000 – FA. Malvern machine. A particle size protocol from the Institute for Ecosystem Research at Kiel University was implemented and adjusted for sediment preparation [25]. To calculate particle size statistics, GRADISTAT (Microsoft Excel) program version 9.1 was used [53]. GRADISTAT uses descriptive terms from [54], which adjusted their particle size classification from the [55] and [56] scales.

The three micromorphology samples were shipped to MK Factory in Stahnsdorf, Germany, for 2—component epoxy resin impregnation and slide mounting. The microscopic analysis of the thin sections was conducted on a Zeiss Axioscope 5 cross-polarised light microscope with ZEN Core 3.5 software. Standardised micromorphological terminology was consulted for descriptive terms, case studies, and mineral identification [57–59].

### 3.3. Optically Stimulated Luminescence

The Optically Stimulated Luminescence (OSL) dating technique relies on the capacity of minerals (i.e., quartz and potassium(K)-rich feldspar) to record the amount of natural radiation to which they have been exposed during burial. In the laboratory, the total amount of energy stored within the mineral since its last exposure to daylight is measured as an equivalent dose (De, Gy). The energy absorption rate (dose rate, Dr, Gy/ka) is derived from knowledge of the natural radioactivity of the sediment. The ratio of the two values (De/Dr) gives the burial age, or age since deposition [60]. In this study, we used OSL dating of quartz and K-rich feldspar at the Luminescence Dating Research Laboratory, Stony Brook University, USA. Full details of the OSL dating of quartz and K-rich feldspar are provided in the Supplementary Information, S2.

### 3.4. Carbonate stable isotopes and plant wax biomarkers

A sub-sample of all homogenised bulk sediment samples (n = 22, whereby only column samples were used in the upper sequence) was measured for carbon and oxygen isotope composition in the Leibniz Laboratory for Radiometric Dating and Stable Isotope Research at Kiel University using a Thermo Scientific MAT253 mass spectrometer connected to an automated carbonate preparation device (Kiel IV). Each sample reacted with 100% H_3_PO_4_ at 75°C under vacuum. The evolved carbon dioxide was analysed eight times per sample. Values are given in δ notation, as opposed to Vienna Pee Dee Belemnite (VPDB). The analytical precision is better ±0.08‰ for δ^18^O and ±0.05‰ for δ^13^C as documented by the performance of international carbonate standards (each pair of isotope values for standards in the order (δ^13^C, δ^18^O): NBS-19 + 1.95‰VPDB, −2.20‰VPDB; IAEA603 + 2.46‰VPDB, −2.37‰VPDB) and laboratory internal carbonate standards (Hela1 + 0.91‰VPDB, + 2.48‰VPDB; SHK + 1.74‰VPDB, −4.85‰VPDB; HB1–12.10‰VPDB, −18.10‰VPDB). A laboratory internal dolomite standard was also analysed (Salv3 + 0.50‰VPDB, −2.96‰VPDB). Samples were measured twice, with a reaction time of 4 minutes and 18 minutes, respectively. The shorter reaction time ensures that the complete reaction of calcite and aragonite occurs. A longer reaction time is needed to allow the dolomite to completely react with coarser grain sizes. Comparable results for the two reaction times demonstrate a complete reaction after the shorter reaction time (see Supplementary Information, S1). Calcite is present in the samples.

Sediment samples for plant wax biomarker extraction (n = 15) were taken directly from the exposed profile at 10 cm intervals with clean trowels and packaged into aluminium foil. All samples were oven-dried at 60°C for 48 hours before being sieved (1 mm mesh) and homogenised by hand using an agate mortar and pestle. *N*-alkanes were extracted and quantified, following a modified protocol from [61]. A maximum of 16 g of sediment was used to extract organics using an Accelerant Solvent Extractor (ASE-350, Dionix) at 100 bar and 100˚C using a 9:1 (v = v) mixture of dichloromethane (DCM) and methanol. The extracted organic solution was filtered through activated silica gel (140−230 mesh) and silver nitrate (AgNO_3_) coated silica gel via column chromatography to isolate *n*-alkanes. The product was dried under a stream of nitrogen gas and transferred into vials for gas chromatography. Individual *n*-alkane homologues were identified with an Agilent 6890N gas chromatograph equipped with a Restek XTI-5 capillary column (30 m x 320 μm x 0.25 μm) based on the comparison of their retention times in comparison to a standard containing *n*-alkane homologues of known concentration, which was measured at the start and end of every batch of six samples. All the samples were measured in triplicates. See the Supplementary Information, S1 for extended methods and calculation of indices.

The δD and δ^13^C values of terrestrial-sourced *n*-alkane homologues of sufficient concentration (i.e., *n*-C27, *n*-C29, *n*-C31, and *n*-C33) were analysed using gas chromatography-isotope ratio mass spectrometry (GC-IRMS) at the Leibniz Laboratory for Radiometric Dating and Isotope Research at Kiel University. Samples were measured on an Agilent 6890 gas chromatograph equipped with a Gerstel KAS 4 PTV injector and an Agilent DB-5 capillary column (30 m x 250 μm x 0.25 μm) coupled to a Thermo Scientific MAT 253 isotope ratio mass spectrometer (IRMS). The following GC temperature program was used for the analysis of carbon or hydrogen isotopes: 50°C for 5 min, with 40°C/min to 240°C, with 20°C/min to 280°C, with 10°C/min to 325°C, 325°C for 20 min Depending on the *n*-alkane concentration, 10.0 μl and 20.0 μl of each sample for δ^13^C and δD, respectively, was injected 2–4 times to achieve a statistically robust analytical error for each *n*-alkane homologue. To allow large volume injections (LVI), the injector was used in solvent vent mode. The H3 + factor during the measurement period was 10.25 ± 0.23 ppm/nA (*n* = 18). Using Arndt Schimmelmann’s A7 reference mixture from 2017, the δD and δ^13^C values are reported relative to Vienna Standard Mean Ocean Water (‰ VSMOW) and Vienna Pee Dee Belemnite (‰ VPDB) scales, respectively.

Additional information regarding the ethical, cultural, and scientific considerations specific to inclusivity in global research is included in the Supporting Information (S4 Checklist). All necessary Fs were obtained for the described study, which complied with all relevant regulations. Fieldwork was conducted under research permit ENT 8/36/4 L (56), issued on 26 August 2021 by the Botswana Ministry of Environment, Wildlife and Tourism.

## 4. Results

### 4.1. Field descriptions

The lower section of MAR-T4 (124–169 cm) consists of consolidated (duricrust) lacustrine sediment (Fig 2). This section is characterised by indurated silty/clayey sediment. Heavy bioturbation by plant roots and mesofauna was noted throughout the profile. The sediment from 94–124 cm is slightly less indurated than that in the section below. This portion of the sequence includes a sandy component which fills the cracks that were formed by cycles of hydration and desiccation. The uppermost section, from 0–94 cm, also has similar column and inter-column structures, although the duricrust is less cemented than below. The sediments from 64 cm below the surface up to the surface contain small pieces of incipient duricrust and show signs of heavy bioturbation, with the layer between 40–50 cm being particularly heavily impacted by bioturbation processes. The upper 0–30 cm below the surface is made up of a layer of silty, unconsolidated sand with small (< 2 cm) duricrust nodules (Fig 2).

### 4.2. Bulk sediment results

**Fig 3:**
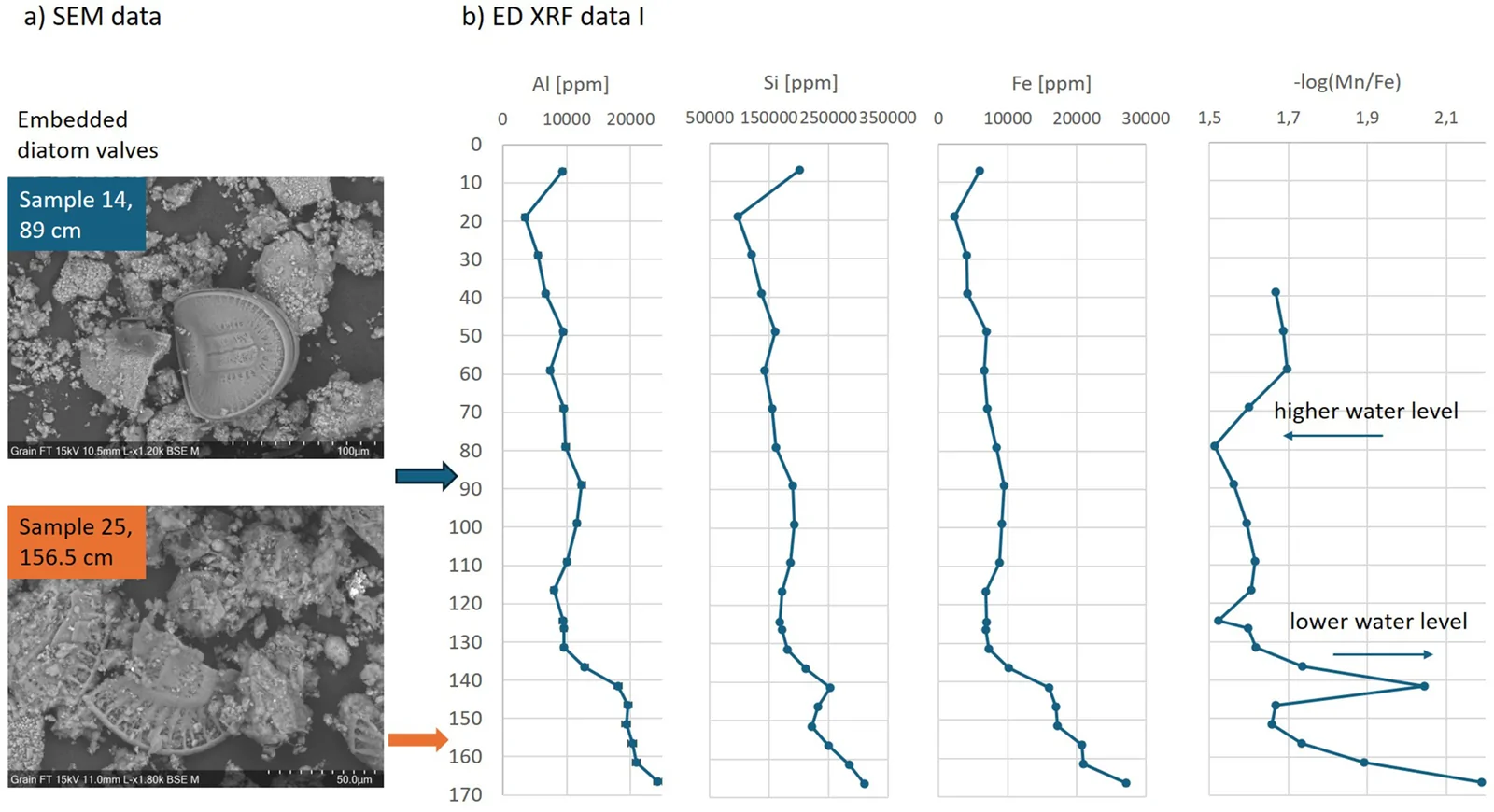
a) SEM data revealing the presence of diatoms (*Campylodiscus* sp.) over large parts of the lower sequence, b) ED-XRF data displaying some major elements of the sequence and the Mn/Fe ratio indicative of water depth (only “column” samples).

Diatoms (*Campylodiscus* sp.) were identified using an SEM in samples taken at depths 84–94 cm (MAR-T4.14) and 149–154 cm (MAR-T4.25) below the surface (Fig 3). This taxon was also identified in other duricrust profiles at MAR [25]. No diatom remains were observed in samples from the upper 50 cm of MAR-T4.

**Fig 4:**
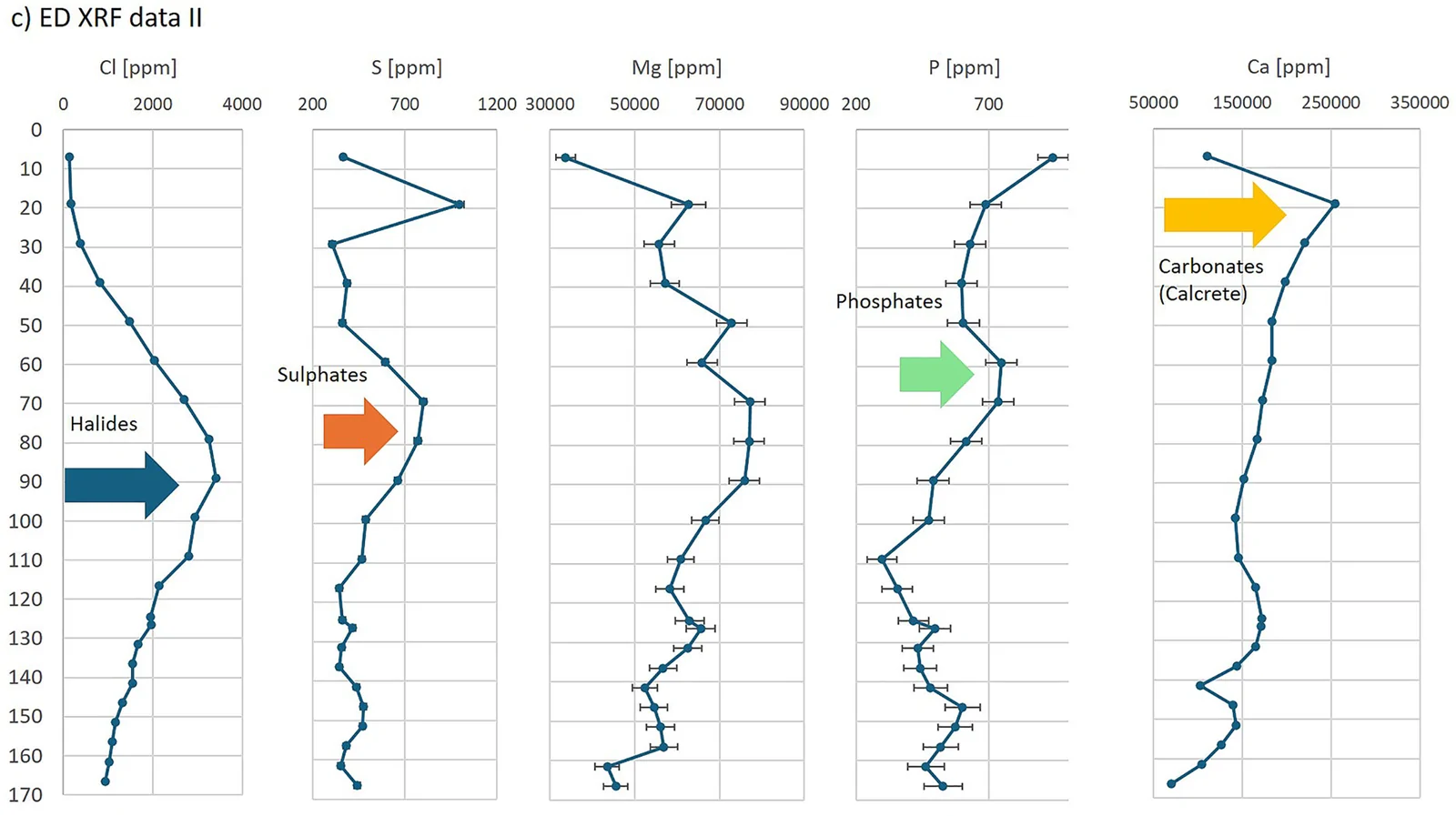
Displaying ED-XRF data indicative of calcrete formation (only “column” samples).

The ED-XRF results are presented in Figs 3 and 4, and a complete list is provided in Supplementary Information 3 Table S9 in S2 File. The major elements are also given as oxides expressed as percentages for ease of comparison with available literature from other Kalahari duricrusts.

Trends are present in the concentrations of elements relating to silicate minerals (Fig 3). Al, Si, Fe, and also K and Zr peak at the base of the sequence and show a decreasing trend towards the surface.

**Fig 5:**
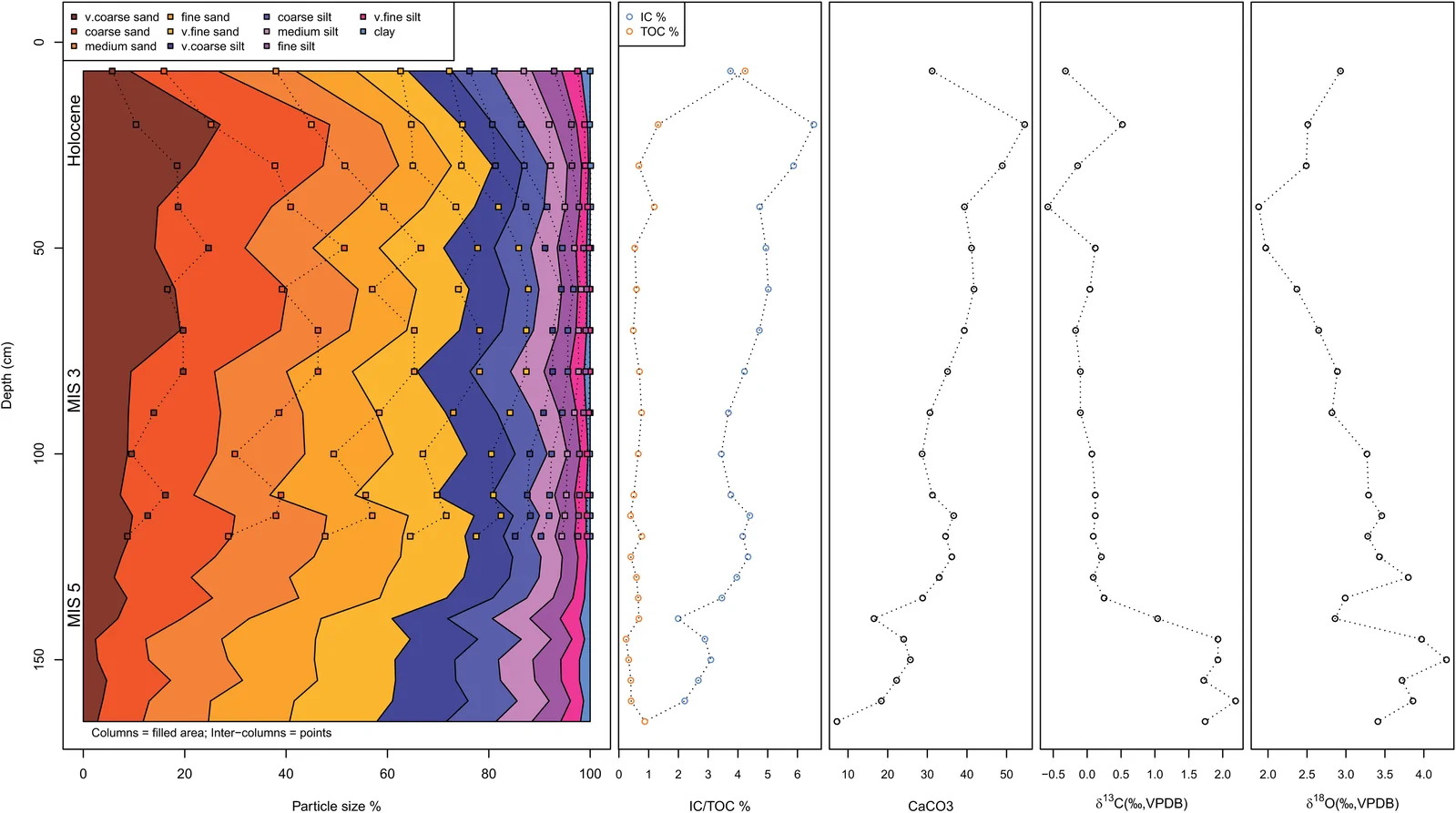
MAR-T4 sequence with the particle size distributions, TOC% and IC%, CaCO_3_ concentrations, and carbonate stable isotope results.

The results indicate a decline in CaCO_3_ content with depth (from 31.23% to 7.21%), albeit not a direct linear decrease (Table 1; Fig 5). CaCO_3_ is most abundant in the upper 119 cm of the profile (the highest concentration is 54.6% at 14–24 cm), thus indicating that this portion of the sequence can be classified as a calcrete. A similar trend to the CaCO_3_ results can be observed in the pED-XRF Ca values. MAR-T4.6 (Columns) at 0–14 cm has the highest TOC% content (Table 1). TOC% values decrease towards the lower part of the sequence. The XRD analysis identified only calcite and quartz in the samples analysed (Supplementary Information 1 S8A Fig—H and Table S13 in S2 File).

**Table 1:** Geochemistry and carbonate stable isotope results for all MAR-T4 samples. TOC and TIC are presented as percentages. Stable isotope results are reported against VPDB in per mill (‰). The sediment classification is based on geochemistry and field descriptions.

Sample name	Depth below surface	IC%	TOC %	CaCO_3_%	δ^13^C	δ^18^O	Classification
MAR-T4.6 Inter-columns	0-14 cm	N/A	N/A	N/A	N/A	N/A	Unconsolidated sand with duricrust nodules.
MAR-T4.6 Columns	0-14 cm	3.8	4.2	31.2	−0.3	2.9	Incipient calcrete
MAR-T4.7 Inter-columns	14-24 cm	N/A	N/A	N/A	N/A	N/A	Unconsolidated sand with duricrust nodules.
MAR-T4.7 Columns	14-24 cm	6.6	1.3	54.6	0.5	2.5	Incipient calcrete
MAR-T4.8 Inter-columns	24-34 cm	N/A	N/A	N/A	N/A	N/A	Unconsolidated sand with duricrust nodules.
MAR-T4.8 Columns	24-34 cm	5.9	0.7	48.9	−0.1	2.5	Incipient calcrete
MAR-T4.9 Inter-columns	34-44 cm	N/A	N/A	N/A	N/A	N/A	Unconsolidated sand with duricrust nodules.
MAR-T4.9 Columns	34-44 cm	4.7	1.2	39.4	−0.6	1.9	Calcrete
MAR-T4.10 Inter-columns	44-54 cm	N/A	N/A	N/A	N/A	N/A	Unconsolidated sand with duricrust nodules.
MAR-T4.10 Columns	44-54 cm	4.9	0.5	41.2	0.1	2.0	Calcrete
MAR-T4.11 Inter-columns	54-64 cm	N/A	N/A	N/A	N/A	N/A	Unconsolidated sand with duricrust nodules.
MAR-T4.11 Columns	54-64 cm	5.0	0.6	41.8	0.0	2.4	Calcrete
MAR-T4.12 Inter-columns	64-74 cm	N/A	N/A	N/A	N/A	N/A	Unconsolidated sand with duricrust nodules.
MAR-T4.12 Columns	64-74 cm	4.7	0.5	39.3	−0.2	2.6	Calcrete
MAR-T4.13 Inter-columns	74-84 cm	N/A	N/A	N/A	N/A	N/A	Unconsolidated sand with duricrust nodules.
MAR-T4.13 Columns	74-84 cm	4.2	0.7	35.1	−0.1	2.9	Calcrete
MAR-T4.14 Inter-columns	84-94 cm	N/A	N/A	N/A	N/A	N/A	Unconsolidated sand with duricrust nodules.
MAR-T4.14 Columns	84-94 cm	3.7	0.8	30.7	−0.1	2.8	Calcrete with diatoms
MAR-T4.15 Inter-columns	94-104 cm	N/A	N/A	N/A	N/A	N/A	Unconsolidated sand with duricrust nodules.
MAR-T4.15 Columns	94-104 cm	3.4	0.7	28.7	0.1	3.3	Calcrete
MAR-T4.16 Inter-columns	104-114 cm	N/A	N/A	N/A	N/A	N/A	Unconsolidated sand with duricrust nodules.
MAR-T4.16 Columns	104-114 cm	3.8	0.5	31.3	0.1	3.3	Calcrete
MAR-T4.17 Inter-columns	114-119 cm	N/A	N/A	N/A	N/A	N/A	Unconsolidated sand with duricrust nodules.
MAR-T4.17 Columns	114-119 cm	4.4	0.4	36.7	0.1	3.5	Calcrete
MAR-T4.18 Inter-columns	119-124 cm	N/A	N/A	N/A	N/A	N/A	Unconsolidated sand with duricrust nodules.
MAR-T4.18 Columns	119-124 cm	4.2	0.8	34.6	0.1	3.3	Calcrete
MAR-T4.19	124-129 cm	4.3	0.4	36.2	0.2	3.4	Calcrete
MAR-T4.20	129-134 cm	4.0	0.6	33.0	0.1	3.8	Calcrete
MAR-T4.21	134-139 cm	3.5	0.7	28.8	0.3	3.0	Calcrete
MAR-T4.22	139-144 cm	2.0	0.7	16.6	1.0	2.9	Calcrete
MAR-T4.23	144-149 cm	2.9	0.2	24.1	1.9	4.0	Calcrete with diatoms
MAR-T4.24	149-154 cm	3.1	0.3	25.7	1.9	4.3	Calcrete
MAR-T4.25	154-159 cm	2.7	0.4	22.3	1.7	3.7	Calcrete with diatoms
MAR-T4.26	159-164 cm	2.2	0.4	18.4	2.2	3.9	Calcrete
MAR-T4.27	164-169 cm	0.9	0.9	7.2	1.7	3.4	Calcrete

Results for the frequency-dependent magnetic susceptibility are too low to be interpreted. This is likely due to low iron and titanium concentrations in the sediment. The values are given in the supplementary online material (Supplementary Information 3; Tables S11 and S12 in S2 File).

Particle sizes are presented as percentages in Fig 5, and the GRADISTAT classification is presented in S3, Table S10 in S2 File in the Supplementary Information. Most of the samples from MAR-T4 are poorly to very poorly sorted. Unimodal distributions dominate the lower part of the sequence, up to sample MAR-T4.16 (below 114 cm). Above 114 cm, distributions range from unimodal to bimodal. The sequence is dominated by the coarse sand fraction (1 mm-500 µm); however, below MAR-T4.18 (124 cm), the sediments become finer and are dominated by medium sand (500−250 µm), fine sand (250−125 µm), and very fine sand (125−63 µm). The inter-column samples tend to have a higher coarse sand fraction than the column samples with depth. The lowermost (MAR-T4.21 to MAR-T4.27; 134 cm to 169 cm) and uppermost (MAR-T4.6 to MAR-T4.8; 0–34 cm) sections contain more clay than the rest of the sequence.

### 4.3. Optically Stimulated Luminescence

The present chronological study is based on coarse (185–255 µm) quartz and K-rich feldspar grains, present in large quantities in all of the samples. The final age results increase with burial depth as shown in Tables 2 and 3. For sample SB155, located at the base of the sequence, quartz multigrain OSL did not yield a reliable age due to signal saturation. In contrast, K-rich feldspar provided consistent results, with pIR-IR_225_ and pIR-IR_290_ ages of 101.0 **±** 7.3 ka and 114.1 ± 8.5 ka, respectively, which are statistically indistinguishable at one sigma. For the overlying sample, SB156, the pIR-IR_225_ age is 31.0 ± 3.8 ka, and the multi-grain OSL age is 32.4 ± 2.7 ka, again statistically consistent within uncertainty.

**Table 2:** Summary of equivalent doses and ages of K-rich feldspars at MAR-T4.

SB#	Depth (m)	pIR-IR_225_				pIR-IR_290_			
		n^a^	De^b^ (Gy)	OD^b^ (%)	Age^c^ (ka)	n^a^	De^b^ (Gy)	OD^b^ (%)	Age^c^ (ka)
SB153	0.42	11(11)	27.1 ± 3.9	46 ± 11	9.0 ± 1.4	–	–	–	–
SB156	0.87	12(12)	116.1 ± 12.5	34 ± 8	31.0 ± 3.8	–	–	–	–
SB155	1.45	12(12)	311.2 ± 11.4	9 ± 4	101.0 ± 7.3	12(12)	351.3 ± 14.7	11 ± 4	114.1 ± 8.5

^a^Number of aliquots that passed the selection criteria and were used for the calculation of the De. The total number of aliquots measured is under parentheses.

^b^The final equivalent dose (De) and overdispersion (OD) values were calculated using the Central Age Model (CAM, G [62] using the function calc_CentralDose() v.1.5 from the R ‘Luminescence’ package v.1.0.1 [63,64]. Uncertainties are given at one sigma.

^c^Final age results were calculated using DRAC v.1.2 [65].

**Table 3:** Summary of equivalent doses and ages for Quartz at MAR-T4.

SB#	Quartz Multi-grain				Quartz Single-grain			
	n^a^	De^b^ (Gy)	OD^b^ (%)	Age^c^ (ka)	n^d^	Proportion^e^ (%)	De^e^ (Gy)	Age^c^ (ka)
SB153	12(11)	7.2 ± 1.2	57 ± 12	3.5 ± 0.6	125(354)	58 (FMM1) 20 (FMM2) 22 (FMM3)	3.68 ± 0.1 11.12 ± 0.5 43.34 ± 1.49	1.8 ± 0.1 5.4 ± 0.4 21.1 ± 1.4
SB156	12(12)	90.2 ± 5.2	18 ± 5	32.4 ± 2.7	–	–	–	–
SB155	- (3)	Saturated	–	–	–	–	–	–

^a^Number of aliquots that passed the selection criteria and were used for the calculation of the De. The total number of aliquots measured is under parentheses.

^b^The final equivalent dose (De) and over dispersion (OD) values were calculated using the Central Age Model (CAM [62], using the function calc_CentralDose() v.1.5 from the R ‘Luminescence’ package v.1.0.1 [63,64]. Uncertainties are given at one sigma.

^c^Final age results were calculated using DRAC v.1.2 [65].

^d^Number of grains that passed the selection criteria and were used for the calculation of the De. The total number of grains measured is under parentheses.

^e^The final equivalent dose (De) was calculated using the Finite Mixture Model (FMM [62],) using a three component model using the function calc_FiniteMixture() v.04.3. from the R ‘Luminescence’ package v.1.0.1 [63,64]. The resulting De is from the FMM component 1 that best represents the primary peak of the De distribution; uncertainty is given at one sigma.

### 4.4. Carbonate stable isotopes and plant wax biomarkers

Six carbonate stable isotope sediment samples needed 18 minutes for the complete reaction of the carbonate minerals present in the samples. For these samples, the results of the longer reaction time are used, while in all other cases, the average isotope values of both reaction times are used (see Supplementary Information 3, Table S6 and S2 File for full results). Sample MAR-T4.9 has the lowest δ^13^C and δ^18^O value, while samples towards the lower part of the profile have the highest δ^13^C and δ^18^O values. However, δ^13^C and δ^18^O values do not always follow the same trend. Overall, the δ^13^C values range from a minimum of −0.6‰ to a maximum value of 2.2‰, with the average of 0.5‰. The δ^18^O values range from a minimum of 1.9‰ to a maximum value of 4.3‰, with an average of 3.1‰.

**Fig 6:**
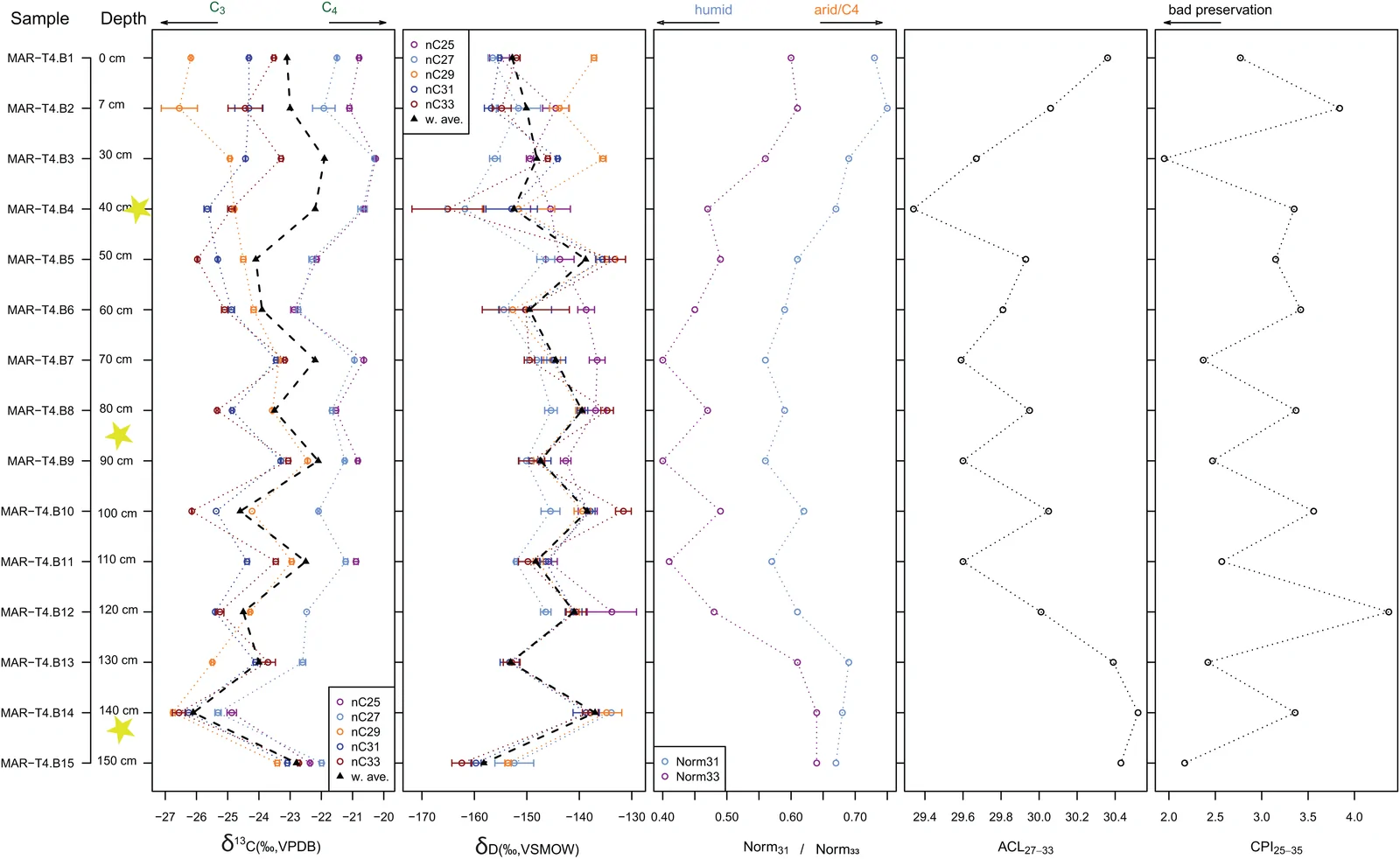
Leaf wax n-alkane results for MAR-T4. From left right: Sample name and depth below surface in cm; δ^13^C isotope values and their weighted average; δD isotope values and their weighted average; *Norm*31 and *Norm*33 index values; Average chain length (ACL) of the C_27_-C_33_ n-alkanes; Carbon Preference Index (CPI) of the C_25_-C_35_ n-alkanes. Yellow stars mark the location of the three luminescence dating samples.

The plant biomarker samples show good preservation for *n*-alkanes with no evidence of substantial degradation of *n*-alkane chains [66,67]. Indicators for good preservation are the odd-over-even ratio with clear odd dominance (Supplementary Information 3 Table S7 in S2 File) and carbon preference index (CPI) values above 2 (Table 4). The concentration of *n*-alkanes in the samples was variable but high enough for stable isotope measurements (Supplementary Information S1). Higher chain lengths dominate in most samples, indicating a terrestrial plant wax source with average chain length (ACL_27 − 33_) ranging from 29.3 to 30.5 (average 30.0 ± 0.4, n = 16) (Table 4; Fig 6). The small variation in ACL is also reflected in the dominant homologue, which is C_31_ in all samples except for three samples, in which C_25_ is the dominant homologue (Table 4). The carbon preference index (CPI) of the C_25_ − C_35_ *n*-alkanes ranges between 2.0 and 4.4 (average 3.0 ± 0.7, n = 16) (Table 4). The *Norm*31 (average 0.6 ± 0.1) and *Norm*33 (average 0.5 ± 0.1) show the most variation in the uppermost and lowermost samples (Table 4; Fig 6).

**Table 4:** Leaf wax *n*-alkane indices and stable isotope results for MAR-T4. From left to right: Sample name and depth below surface in cm; *Norm*31 and *Norm*33 index; Average chain length (ACL) of the C_27_-C_33_ *n*-alkanes; Carbon Preference Index (CPI) of the C_25_-C_35_ *n*-alkanes; δ^13^C isotope values (‰ VPDB); δD isotope values (‰ VSMOW).

Sample	Depth	*Norm*_31_	*Norm*_33_	ACL_27–33_	CPI_25–35_	δ^13^C nC_25_	δ^13^C nC_27_	δ^13^C nC_29_	δ^13^C nC_31_	δ^13^C nC_33_	δD nC_25_	δD nC_27_	δD nC_29_	δD nC_31_	δD nC_33_
MAR-T4.B1	top	0.73	0.60	30.4	2.8	−20.8	−21.5	−26.2	−24.3	−23.5	−155.2	−156.5	−137.2	−155.2	−152.0
MAR-T4.B2	7 cm	0.75	0.61	30.1	3.8	−21.1	−21.9	−26.6	−24.3	−24.4	−144.5	−151.6	−143.8	−156.8	−154.8
MAR-T4.B3	30 cm	0.69	0.56	29.7	2.0	−20.3	−20.3	−24.9	−24.4	−23.3	−149.4	−156.1	−135.5	−144.1	−146.0
MAR-T4.B4	40 cm	0.67	0.47	29.3	3.4	−20.6	−20.7	−24.8	−25.7	−24.9	−145.5	−161.8	−151.6	−152.9	−165.1
MAR-T4.B5	50 cm	0.61	0.49	29.9	3.2	−22.2	−22.3	−24.5	−25.3	−26.0	−143.7	−146.4	−134.8	−135.6	−133.2
MAR-T4.B5	50 cm	0.59	0.45	29.8	3.4	−22.9	−22.7	−24.2	−24.9	−25.1	−138.7	−154.4	−152.7	−150.3	−150.2
MAR-T4.B6	60 cm	0.56	0.40	29.6	2.4	−20.6	−20.9	−23.4	−23.5	−23.2	−136.6	−148.1	−145.2	−144.9	−149.5
MAR-T4.B7	70 cm	0.59	0.47	30.0	3.4	−21.5	−21.7	−23.6	−24.9	−25.3	−136.9	−145.4	−140.0	−139.4	−134.7
MAR-T4.B8	80 cm	0.56	0.40	29.6	2.5	−20.8	−21.3	−22.4	−23.3	−23.1	−142.6	−150.1	−147.7	−147.5	−149.1
MAR-T4.B9	90 cm	0.62	0.49	30.1	3.6		−22.1	−24.	−25.4	−26.2	−138.4	−145.5	−139.4	−137.9	−131.6
MAR-T4.B10	100 cm	0.57	0.41	29.6	2.6	−20.9	−21.2	−23.0	−24.4	−23.5	−145.9	−152.1	−147.8	−146.4	−149.8
MAR-T4.B11	110 cm	0.61	0.48	30.0	4.4		−22.5	−24.3	−25.4	−25.3	−133.8	−146.4	−141.3	−141.1	−140.6
MAR-T4.B12	120 cm	0.69	0.61	30.4	2.4		−22.6	−25.5	−24.1	−23.7				−153.3	−152.9
MAR-T4.B13	130 cm	0.68	0.64	30.5	3.4	−24.9	−25.3	−26.8	−26.3	−26.6		−133.9	−134.8	−138.7	−137.9
MAR-T4.B14	140 cm	0.67	0.64	30.4	2.2	−22.4	−22.0	−23.4	−23.1	−22.7		−152.4	−153.6	−159.7	−162.4
MAR-T4.B15	150 cm	0.73	0.60	30.4	2.8	−20.8	−21.5	−26.2	−24.3	−23.5	−155.2	−156.5	−137.2	−155.2	−152.0

The carbon and hydrogen stable isotope results for C_25_, C_27_, C_29_, C_31_ and C_33_ are given in Table 1, and the full results can be found in the Supplementary Information Table S6 in S2 File. The δ^13^C results exhibit comparable isotopic signatures throughout the sequence with the C_25_ and C_27_ *n*-alkanes displaying the highest δ^13^C values. The δ^13^C values are on average −21.5 ± 1.3‰ for C_25_ and −21.9 ± 1.1‰ for C_27_, but range from −22.4‰ to −26.8‰ in the C_29_, C_31_ and C_33_ homologues. The *n*-alkane chain lengths are not correlating with the hydrogen isotope values, with C_25_ or C_29_ showing the lowest δD values. The δD values range from –133.8‰ to –155.2‰ in C_25_, and from –131.6‰ to –165.1‰ in C_33_, with similar averages but similarly wide variation within each homologue of –143.6 ± 6.8‰ (C_25_), −150.5 ± 6.7‰ (C_27_), −142.8 ± 6.6‰ (C_29_), −147.7 ± 7.6‰ (C_31_) and –147.6 ± 9.8‰ (C_33_).

### 4.5. Micromorphology

**Fig 7:**
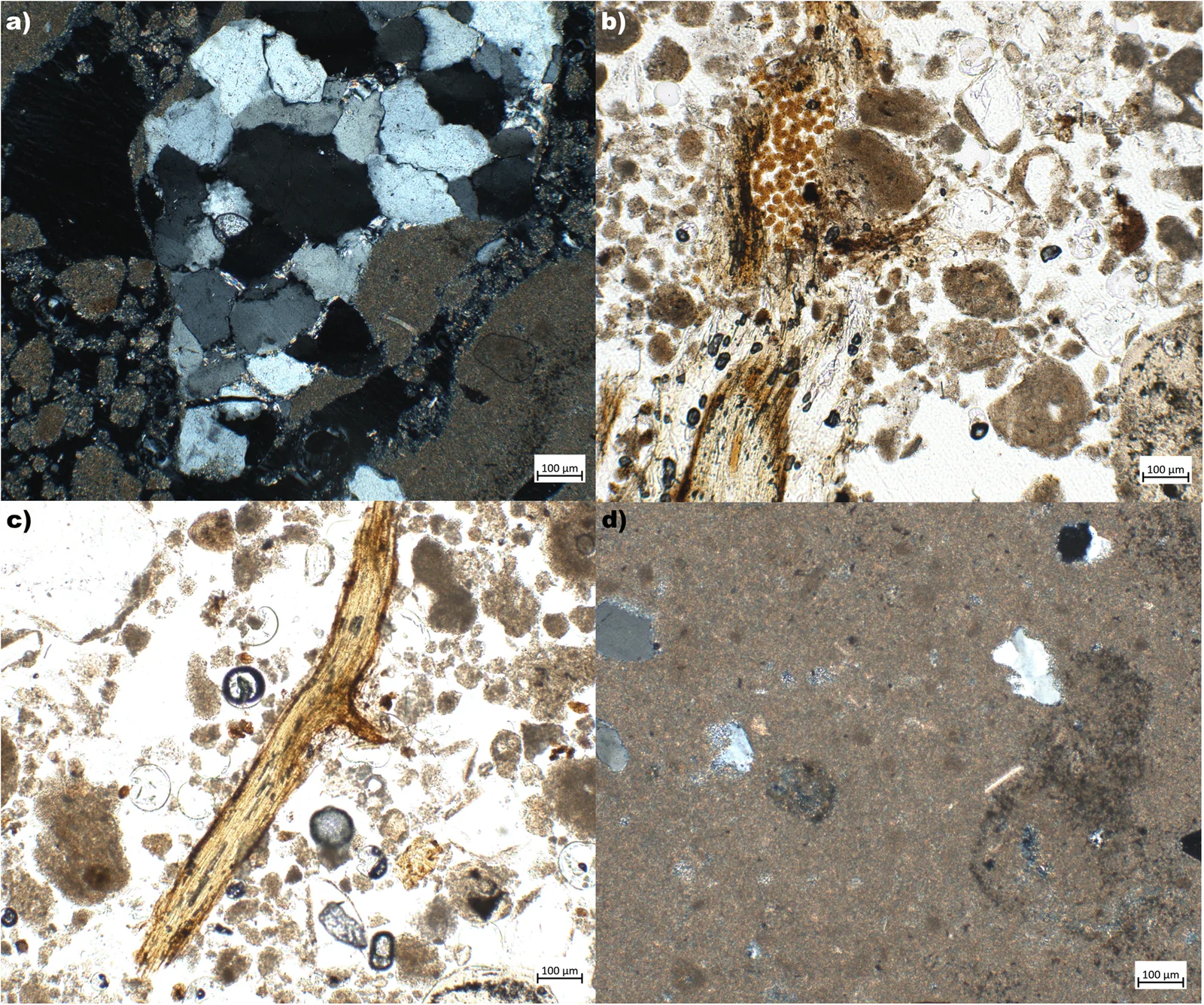
Thin section images of unconsolidated sediment from the upper section of MAR-T4 (38 cm below the surface, sample MAR-T4. M4) a) Quartz being fragmented by secondary calcite crystal growth b) mesofauna (insect) excrement inside a root structure c) a longitudinal section of a root d) sub-angular quartz grains with diffused edges inside a calcrete pebble.

Limited micromorphological features could be described for MAR-T4.M4 and MAR-T4.M1, as these samples consisted of loose sediment that was at risk of being moved around in the Kubiena box prior to resin impregnation. However, we still describe the identifiable content. The scans of the full slides are provided in the Supplementary Information (S9 Fig). In MAR-T4.M4, secondary calcite crystal growth inside quartz grains (Fig 7a), bioturbation (Fig 7b and c) and weathering of quartz grains (Fig 7d) are observed. The particle size distribution is poorly sorted to very poorly sorted in the thin section and in the Mastersizer results (Fig 7a and d; Supplementary Information Table S10 in S2 File).

**Fig 8:**
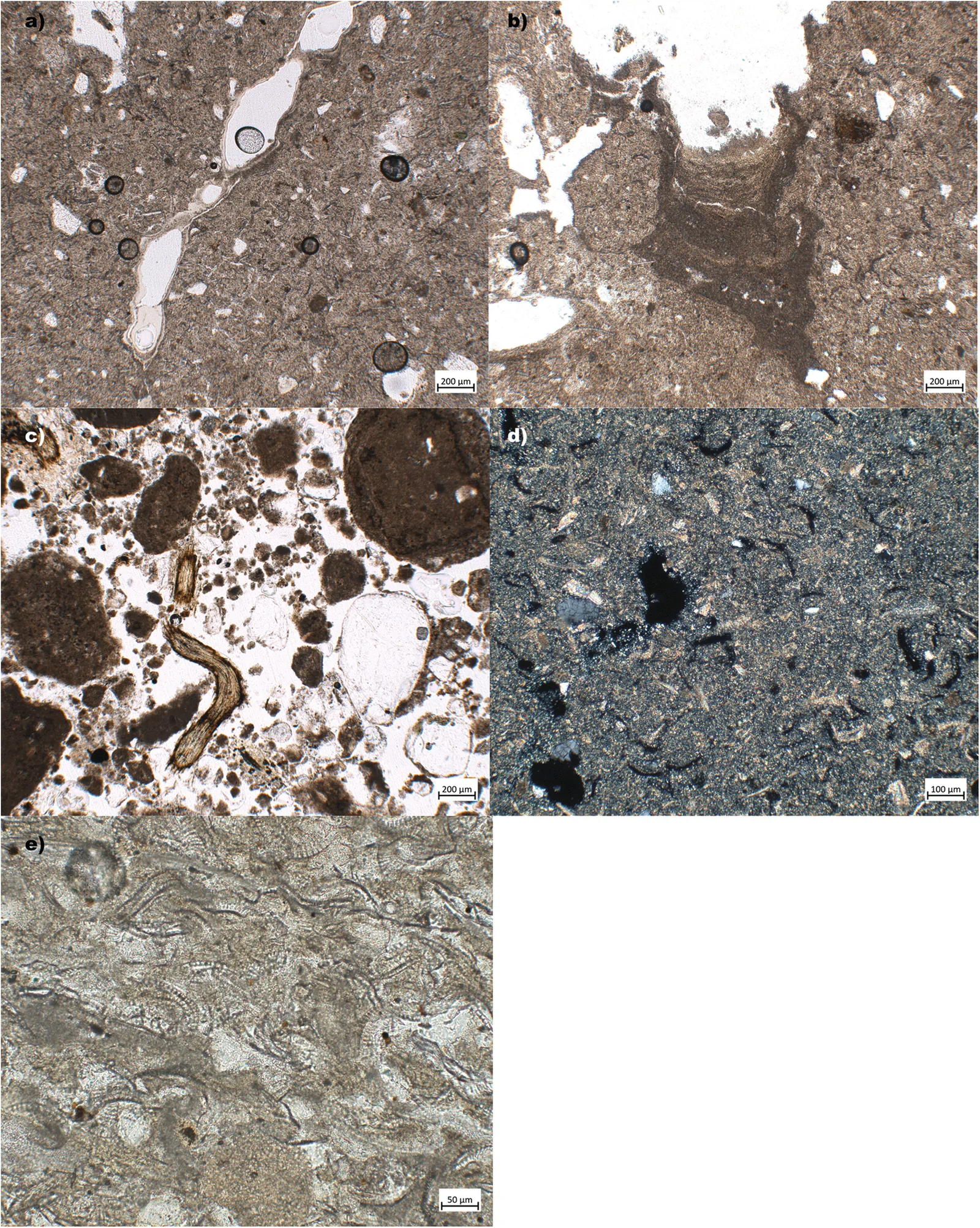
Thin section images of unconsolidated sediment taken 90 cm below the surface in MAR-T4 (sample MAR-T4. M1). a) clay coating of a channel void b) bioturbation by burrowing mesofauna c) longitudinal root debris d) a quartz grain with diffused edges, suggesting in situ weathering e) diatom remains in a micritic micromass.

In MAR-T4.M1, the presence of calcite nodules allows for the identification of a crystallitic b-fabric (birefringent calcite). The sediment has a poorly sorted particle size distribution (Fig 8; Supplementary Information Table S10 in S2 File). Clay void coatings are observed (Fig 8a). An insect burrow (Fig 8b) and roots (Fig 8c) suggests that bioturbation has affected the sediment. The quartz pieces with diffused edges indicate *in-situ* weathering by secondary crystal growth (Fig 8d). Diatom remains are present in some of the more micritic sections (Fig 8e).

**Fig 9:**
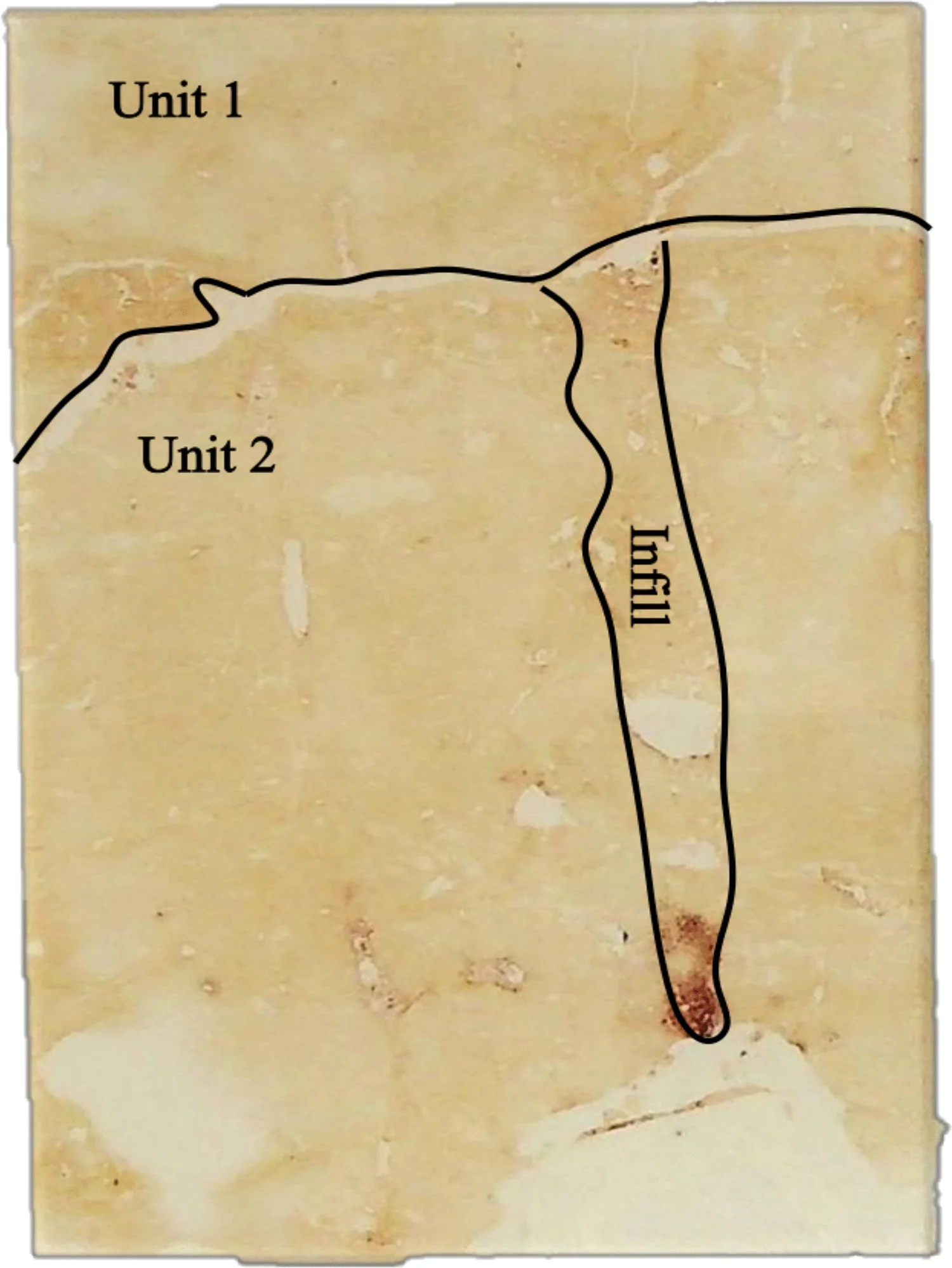
The scan of MAR-T4. M3 dividing the thin section into three units – Unit 1, Unit 2, and an infill unit. Planar voids and channels are visible on the thin section.

**Fig 10:**
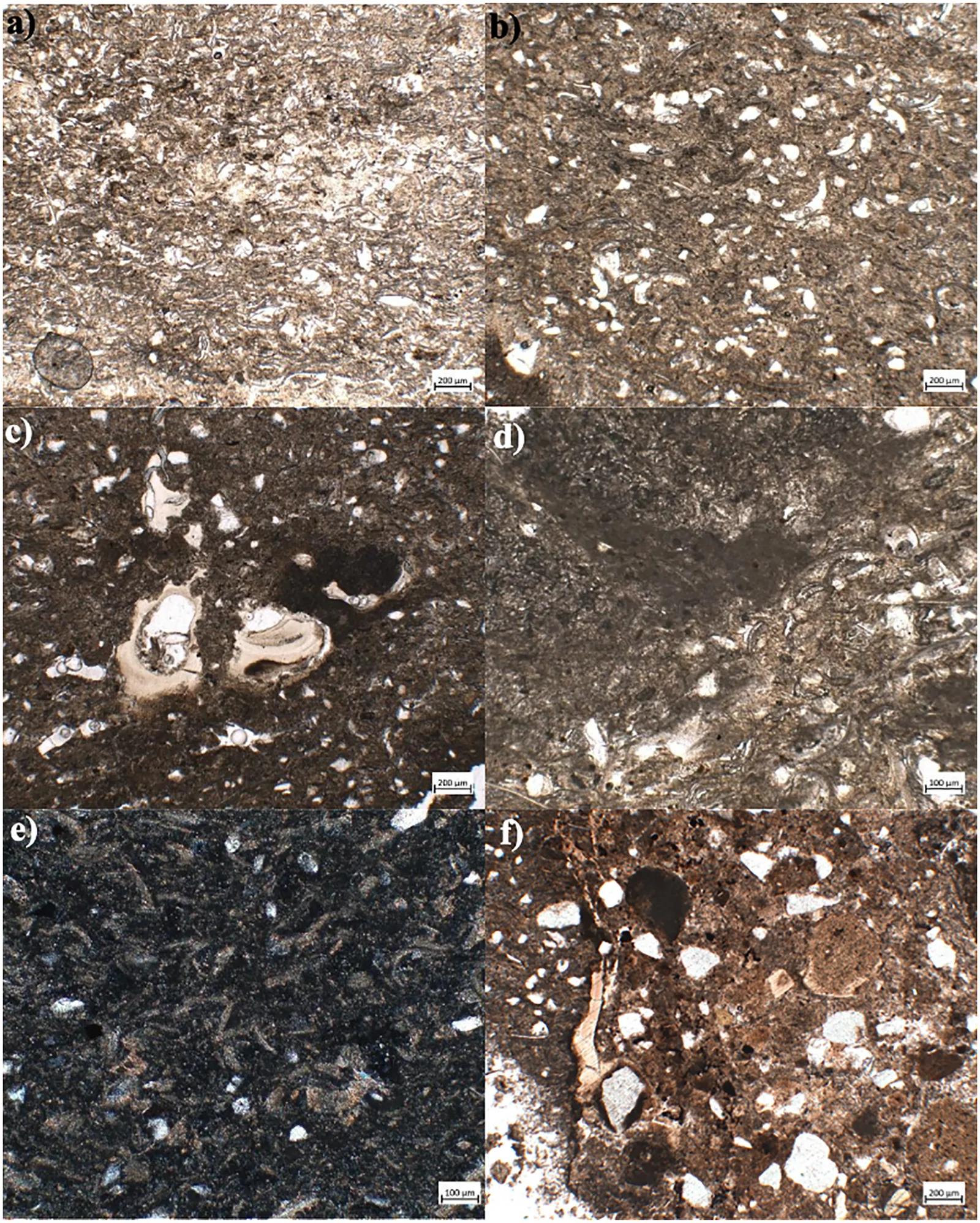
XPL and PPL images of thin section images of MAR-T4. M3. a) Unit 1: a micritic cement (calcrete) b) Unit 2: a coarser micritic cement with floating quartz grains c) Crescentic clay coatings d) Diatoms, microfossil remains, calcite precipitation e) rounded quartz grains with diffused edges in Unit 2 f) Clay particles and sub-rounded quartz grains in the infill unit.

The lowermost micromorphology sample, MAR-T4.M3, has been divided into an upper unit (Unit 1) and a lower unit (Unit 2), which are separated by a planar void (Fig 9). A large section of red sediment infill can also be observed macroscopically. Channels and chambers are observed in Unit 2 (Fig 9). The section has a crystallitic b-fabric throughout, interrupted only in areas of carbonate depletion, as indicated by an almost isotropic micromass. The abundance of diatom and other microfossil remains is striking. An open-porphyric c/f related distribution is present throughout most of the thin section (Fig 10a-b). Features suggesting clay translocation (Fig 10c) and pedofeatures (e.g., waterlogging features, depletion, and desiccation planes) are observed mostly in Unit 2 (Fig 10d). Clay coatings and infillings show a faint whitish-grey colour under XPL and appear almost isotropic in certain areas. Quartz dissolution is illustrated in Fig 10e. An infill with coarser quartz grains is observed in Fig 10f.

## 5. Discussion

The data presented here are indicative of a fluctuating Late Pleistocene environment at MAR-T4. Although the conditions during MIS 5c-e were more similar to today, later periods during MIS 3 were characterised by more humid conditions and possible waterlogging. This was followed by the desiccation of the pan during the Holocene.

### 5.1. Stratigraphy and formation of MAR-T4

The base of the MAR-T4 sequence dates to MIS5c-e, the last interglacial, while the middle part of the sequence records deposition during the Late Pleistocene, during MIS 3.

For the uppermost sample, however, the depositional history appears more complex. The feldspar pIR-IR_225_ age is 9.0 ± 1.4 ka with an OD of ~46%, suggesting partial bleaching and/or post-depositional mixing. Quartz multi-grain OSL yields a younger age of 3.5 ± 0.6 ka with an OD of 57%, consistent with our previous interpretation. The SG quartz results show an even more scattered distribution, with an OD of 100%, with a FMM resolving three components: a dominant young population at 1.8 ± 0.1 ka (58%) and two minor groups at 5.4 ± 0.4 ka (20%) and 21.1 ± 1.4 ka (22%). Taken together, these results point to a stratigraphic unit that has experienced significant post-depositional disturbance. The mixture of age components suggests that while most grains were reset during a recent event (~2 ka), a substantial minority retains older signals from earlier episodes of sediment accumulation or exposure. The discrepancy between feldspar and quartz protocols further underlines the likelihood of incomplete bleaching at deposition, compounded by bioturbation or sediment mixing. Importantly, this complexity is mirrored in the radioelement data, showing a disequilibrium in the head of the U-chain, mainly reflecting geochemical disturbance linked to Late Holocene surface processes. Taken together, this strongly suggests that the uppermost deposits are a palimpsest reflecting multiple episodes of sediment input and reworking through the Late Holocene (MIS 1) in an environment prone to uranium mobility, calcrete formation, and surface disturbance. Further details of the OSL dating of quartz and K-rich feldspar are provided in the Supplementary Information (S2).

The Luminescence results support the other methodologies used, suggesting various formation processes throughout the MAR-T4 sequence. The Mn and Fe ratio in the ED-XRF results (Fig 3) reveals changes in the water level in the pan over time (e.g., [68]), with lower levels during MIS 5 and higher levels during MIS 3, as dated by our OSL results (see section 4.3 and Supplementary Information S2). The mobilisation of carbonates increases when precipitation is higher [69]. The concentrations of Cl, S, P, and Ca follow a depth-related solubility pattern of different precipitation products (halides, sulphates, phosphates, carbonates). The decreasing trend of elements associated with silicate minerals partly reflects dilution resulting from alteration and the addition of evaporative minerals in the upper part of the deposited lake sediment. The elemental results (Fig 4) indicate the formation of incipient calcrete in the upper section (confirming the field observations), suggesting a drying phase of the pan in the mixed Holocene section.

The XRD results (Supplementary material S8a-h Fig) indicate the presence of calcite and quartz in the MAR-T4 profile. The TIC% results (converted to CaCO_3_ by stoichiometric conventions) further confirm that the MAR-T4 sequence is a calcrete profile, with some episodes of increased silica input (most notably in the lower sections of the profile). The abundance of diatoms in the MIS 5c-e and MIS 3 thin sections indicates increased moisture availability in the environment compared to the Holocene layers, where no diatoms were identified. The diatom taxon, *Campylodiscus* sp., suggests the presence of a saline to brackish water body. No statistical analysis of the diatom population has been conducted and palaeoenvironmental inferences from the microfossil assemblages should therefore be approached with caution.

The presence of clay translocation features, signs of bioturbation (biogenic features), the weathering of quartz grains by crystal growth, and a subtle decrease in particle sizes from the upper to the lower sections of the profile suggests mostly pedogenic development of the MAR-T4 profile. Clay translocation, the movement of clay down through a soil profile by percolating water (see [58]), has been linked to pedogenic processes in other semi-arid areas in the southern African interior (e.g., [70]). The formation of an orthic carbonate nodule from Unit 2 in the thin section from MAR-T4.M3 further indicates pedogenesis. Due to the presence of some ‘floating’ quartz grains (Fig 10e), groundwater input is also plausible in the lowermost part of Unit 2. Waterlogging is plausible, as indicated by features such as the depletion of carbonate and whitish-grey (XPL) clay coatings, which are common in hydromorphic sediment. The alternation of water levels is evident from desiccation planes, which suggest increased evaporation. Sub-angular or sub-rounded quartz grains have been identified in both MIS 5 and MIS 3 thin sections, but the sediment is volumetrically dominated by a fine, micritic cement in both cases. Since quartz grains were weathered by secondary crystal growth, grain size and shape have likely been altered from their state at the time of deposition. The modern Kalahari Sands in the profile (represented by inter-column samples) filtered down from upper layers through desiccation cracks. Sediment cycling in the pan environment has mostly affected the upper sediments, which are characterised by mixed Holocene deposition (Fig 5) (see [32]). These samples are generally poorly sorted, but show evidence of some aeolian input in the thin section, as sub-rounded, moderately sorted quartz grains are present.

### 5.2. Palaeoenvironmental signal at MAR-T4

Plant waxes can be transported by wind or water and usually represent a local or regional environmental signal. Three main site formation processes have previously been identified at MAR Pan, namely runoff, deflation, and duricrust formation [25]. These processes were the main natural drivers of biomarker input into the sediments within the pan. Some plant groups produce greater amounts of plant waxes than others, which might skew the representation towards these plant groups [71–73]. Plants could also have been brought into the site by humans. However, there are very few artefacts and no evidence of intense human use of the MAR-T4 site.

The lowermost plant biomarker samples (B13-B15; 130–150 cm) in MIS 5c-e have *Norm*31, *Norm*33 and ACL_27–33_ indices in the same range as the uppermost Holocene samples. This implies an arid environment with plants experiencing water scarcity during MIS 5c-e, with a summer rainfall regime similar to today. Carbonate oxygen isotope values are the highest in the sequence below 140 cm depth, further supporting a highly evaporative environment during parts of MIS 5c-e.

High C_31_ and C_33_ abundances in these lowermost samples suggest a high proportion of C_4_ plants in the local environment during this time. The TOC% content for this section is consistently low (between 0.87–0.24%), indicating lower biomass accumulation and vegetation density compared to upper samples thus also suggesting more arid conditions (see [50]). Biomarker sample B15 (150 cm) has high δ^13^C values, supporting the presence of C_4_ plants, while sample B14 (140 cm) shows the opposite, with some of the lowest δ^13^C values of the sequence. The δD values show the opposite trend, with some of the lowest δD values in sample B15 and high ones in B14. This suggests a shift in rainfall amount or rainfall source, for example varying amounts of rainfall from the Indian and Atlantic Ocean. Although the carbonate stable isotopes represent different influences, compared to the plant wax biomarker isotopes, a change is also detected in the carbonate stable isotope data between the sample at c. 140 cm and the sample at c. 150 cm, which has the highest δ^13^C value of the sequence. This suggests that several phases within MIS 5 are captured in our data. While carbonates have diverse origins in arid environments, our interpretation based on all available data indicates that while it was an overall terrestrial, arid environment with a dry lakebed, significant changes in vegetation and rainfall/hydrology occurred repeatedly during this time. The abundance of diatoms in the MIS 5 micromorphology sample supports the occurrence of periods of high moisture availability.

The second cluster differs notably, as from 50 cm to 120/130 cm depth below the surface, there is little variation in both the plant wax and carbonate isotope data. There are small fluctuations in all stable isotope values and the indices suggesting that these sediments accumulated steadily over a period of time. Not all the samples between 50 cm and 120/130 cm are directly associated with OSL dates, but these sediments fall within the timeframe of MIS 3. The IC% (high CaCO_3_%), and micromorphology (evidenced by a micritic cement) results for this section indicate that calcite is a dominant mineral in the sediment. The TOC% concentrations vary between 0.39–0.77%, which is similar to the MIS 5 TOC% results.

Sample B8-10 (80–100 cm depth) is associated with the OSL sample SB156 (31.0 ± 3.8 ka). The n-alkane homologue abundances, including strong C_23_ and C_25_ homologue signals, suggest a mix of terrestrial and aquatic plants such as submerged macrophytes. C_33_ abundances are lower than in the MIS 5 samples, suggesting less C_4_ plant input than before. The *Norm*31, *Norm*33, and ACL_27–33_ indices indicate less aridity, and the carbonate oxygen isotopes also show lower evaporation than in MIS 5.

The TOC% from the upper Holocene sequence is the highest in the sequence, and ranges between 0.53 and 4.24%, suggesting greater biomass accumulation compared to the Pleistocene samples below (Table 1). Plant biomarker samples B1-B5 (top 40–50 cm) constitute a third cluster in the data. There is a wide spread of δ^13^C and δD values between the individual homologues in Sample B4 (40 cm). Its ACL_27–33_ value, as well as its δ^18^O carbonate value, are the lowest of the sequence, indicating the least arid conditions of the sequence. High C_25_ homologue abundances and low-chain homologues indicate the influence of submerged and/or emerging macrophytes. However, this part of the profile is highly bioturbated and mixed (as indicated by the OSL dating) and the signal is likely influenced by both bioturbation and the mixing of column and inter-column sediments. All samples in the first 40 cm yielded a wide range of different δ^13^C and δD values for the individual homologues C_25_-C_33_, in contrast to the Pleistocene samples. Equally, the carbonate isotope results for the Holocene section vary widely, with no clear trend. The uppermost samples (B1-B2; 0–7 cm) also yielded high C_25_ and C_31_ amounts, again indicating a mix of terrestrial and aquatic plants. The *Norm*31, *Norm*33 and ACL_27–33_ indices indicate relative aridity/water stress, and an increase in C_4_ plants in the Late Holocene.

### 5.3. Regional comparisons

Our palaeoenvironmental record from MAR indicates the presence of a fluctuating lacustrine environment with aridity similar to the present during the course of MIS 5c-e, including both periods of increased moisture availability and drying phases. Fluctuating environments with alternating wet and dry phases during the late Quaternary have been recorded at various sites in the broader Kalahari (for example, in [20,43,74]), but to varying degrees. At the Makgadikgadi Palaeolake in northern Botswana, an analysis of sediment from archaeological sites in Ntwetwe Pan [20], identified one lake high-stand between 128 ± 18 ka and 81 ± 6 ka; and another between 72 ± 5 ka and 57 ± 8 ka, but suggested that the main period of MSA archaeological site formation occurred when the lakebed was dry, sometime before the two lake level high-stands. This suggests that MSA hominins at Makgadikgadi were capable of navigating arid landscapes during MIS 5 and later periods [20–22]. This record broadly corresponds with our inference of a fluctuating environment and a dry pan surface during parts of MIS 5c-e in the MAR-T4 sequence. During this time, hominins were present on the pan floor and discarded early MSA lithics, which were subsequently excavated from test pits MAR-G1 and MAR-G2 (slightly off-centre in the pan on the southern side) [25].

Our results suggest greater moisture availability during MIS 3, which corresponds with results from an analysis of four ridge sites on the western and northern shorelines at the Makgadikgadi Palaeolake, which revealed increased moisture availability at 64.2 ± 2.0 ka, 38.7 ± 1.8 ka and 26.8 ± 1.2 ka [74], roughly bracketing our MIS 3 depositional age of 31.0 ± 3.8 ka. The palaeoshoreline deposits at Ntwetwe Pan demonstrate that a large water body, which appeared and disappeared at various stages, existed at Makgadikgadi [20,21,74]. This likely occurred at MAR Pan as well, albeit at a significantly smaller scale than at the larger Makgadikgadi Palaeolake. Our inference of increased moisture availability during MIS 3 is further supported by an analysis of a pan located adjacent to the site of Tsodilo Hills in northern Botswana. An analysis of mollusc remains from the pan suggests that a permanent waterbody was present in the area between 40−32 ka [75]. At Ga-Mohana Hill in South Africa, human occupation during MIS 5d, late MIS 3, and late MIS 2 occurred alongside tufa formation, indicating water availability at the site during these periods [43]. Our results from MAR Pan therefore roughly reflect other records from Northern Botswana and the southern Kalahari in South Africa during MIS 3.

The only other published plant wax biomarker record from a Kalahari pan is from Omongwa Pan in Namibia. The analysed sediment dates back to the LGM, with the results from deposits dating to 23–19 ka indicative of dry environmental conditions [50]. The TOC% results (0.2–4.2%) from the MAR-T4 sequence are higher than those of Omongwa Pan, which fall within a range of 0.2–1.4%, suggesting a greater biomass input at MAR Pan during MIS 3 than at Omongwa Pan during the LGM.

Omongwa Pan has a high abundance of low-chain homologues, potentially similar to many of the MAR-T4 MIS 3 samples, in one part of the sequence (“Cluster I”). In contrast, their “Cluster II” has higher C_33_ than C_29_ abundances [50], which is similar to the MIS 5 samples and the topmost modern sample at MAR-T4. At Omongwa Pan, this is partly interpreted as representing increased C_4_ plant input in Cluster II by [50], similar to the interpretation of our MIS 5 samples.

A comparison between the MAR-T4 plant biomarker record with the record from the Holocene layers at Wonderwerk Cave in South Africa [76], reveals an overlap in the range of hydrogen stable isotope values, indicating similar origins of the rainfall system. However, MAR-T4 has higher δ^13^C values than Wonderwerk Cave. This is likely due to different plant source regions, with Wonderwerk Cave opening onto the Ghaap Plateau and MAR-T4 being located in a more arid section of the Kalahari.

Also in the South African section of the Kalahari near Wonderwerk, a shift in environmental conditions was identified at the start of the Holocene at Kathu Pan 6, with periods of aridity (high evaporation) alternating with marshy conditions, and a shift from drier conditions to a period of humidity between 4−2 ka [17]. This signifies a shift towards stronger rainfall seasonality during the Holocene for the region [17].

### 5.4. Human presence at MAR

The recovery of lithics belonging to an early MSA typology demonstrates that hominins were present at MAR during MIS 5. Although there are no lithics from the lower part of MAR-T4, evidence of human presence has been established at nearby MAR-G2 (Fig. 1), which has a lens of artefacts (starting at 80 cm below surface) in a layer dating to late MIS 5. A highly fluctuating environment during MIS 5 would have meant a period of aridity, where hominins could use the pan floor. These periods of accessibility alternated with periods of increased moisture availability when parts of the pan were flooded. The interstadial conditions at the start of MIS 3 correspond with a wetter, less evaporative environment with fewer C_4_ plants, indicating that MAR Pan had standing water for prolonged periods. No lithics dating to MIS 3−2, or lithic typologies from the late MSA and early LSA, have been identified at MAR Pan or its surrounding region [27]. This suggests that the pan floor was not used by hominins during MIS 3, likely due to waterlogging.

Sediment mixing and transportation, in addition to bioturbation, complicate the interpretation of the Holocene portion of the sequence. However, an overall trend towards more arid conditions is evident. The Holocene was likely similar to present-day conditions, where the pan fills up with water seasonally, but not for long periods. This matches with very limited, isolated finds of lithics and ostrich eggshell fragments at MAR (in the inner dune test pits MAR-T1 and MAR-T3 (Fig. 1)). The lithics found in MAR-T4 were all excavated from the first 50 cm of the test pit, in the mixed Holocene sediments. Seven flakes and one indeterminate piece were identified, with only one complete piece. One of the flakes has an elongated shape and is likely a laminar flake. Cortex is present on a single artefact, but some of the artefacts have a white coating over large parts of the surface. The condition of the artefacts is indicative of weathering and mechanical wear consistent with transport and post-depositional mixing. The distribution within the test pit suggests that single finds accumulated over a long time span. Four artefacts were recorded in the first 10 cm below the surface, with artefact abundance subsequently decreasing with depth. Two artefacts were excavated between 20–30 cm, and only one artefact per spit was excavated between 30–40 cm and 40–50 cm. The artefacts are light, weighing roughly 23.5 g on average, suggesting that they were transported to this location due to natural processes, such as runoff.

## 6. Conclusion

Assessing the palaeoenvironmental context of Pleistocene deposits in the semi-arid interior of southern Africa has generally been challenging due to low organic preservation and the limited availability of suitable dating methods. The results obtained from sediment analysis of the MAR-T4 sequence, located at the centre of MAR, demonstrate that the environment fluctuated greatly at this site during a period of human presence in MIS 5c-e. Moisture was available seasonally, but cycles of aridity took place throughout this period, with potential shifts in rainfall amount or rainfall systems indicated by plant wax hydrogen isotope abundances. We have provided evidence of increased moisture during late MIS 3, which could have attracted groups of humans to MAR. However, there is currently a lack of archaeological evidence for occupation during this time period. These results show that humans had the ability to adapt to, and navigate, the semi-arid landscape at MAR during the Late Pleistocene. This suggests that proximity to water was not a prerequisite for landscape utilisation by these populations. This is also reflected in other arid sites, such at the Makgadikgadi Palaeolake where inferences have been made that humans were able to adapt to arid environments and likely had the cognitive capacity to move water in containers with them across landscapes [21].

## Supporting information

S1 File Section S1: Biomarkers extended methods. Section S2: Luminescence dating, extended methods and discussion.
(DOCX)

S2 File Tables S6-12. (XLSX)

S3 File Inclusivity in global research. (DOCX)

S1 Fig OSL sampling locations for the MAR-T4 sequence. (TIF)

S2 Fig Assessment of the secular equilibrium in the uranium decay series of sediment samples SB153 (pink), SB156 (blue), SB155 (green), with a comparison between the (A) radioelement concentration derived from the head of the chain and the tail of the chain, and (B) radioelement concentration from the tail of the chain. The 1:1 equilibrium line is indicated by a black line, with dashed lines indicating ± 5%.
(TIF)

S3 Fig Typical feldspar luminescence results for sample SB155: a) Natural pIR-IR_225_ signal, (b) a dose-response curve for the pIR-IR_225_ signal measured on a single aliquot. Regenerated points are shown as full circles. The recycled point is shown as an open circle. The natural signal is shown as a triangle. (c) pIR-IR_225_ dose distribution displayed as a kernel density estimate (KDE) plot. (d) Natural pIR-IR_290_ signal; (e) a dose-response curve for the pIR-IR_290_ signal measured on a single aliquot; (f) pIR-IR_290_ dose distribution displayed as a KDE plot.
(TIF)

S4 Fig Typical multi-grain quartz luminescence results for sample SB153: a) Natural OSL signal, (b) a dose-response curve for the OSL signal measured on a single aliquot. Regenerated points are shown as full circles. The recycled point is shown as an open circle. The natural signal is shown as a triangle. (c) OSL dose distribution displayed as a kernel density estimate (KDE) plot.
(TIF)

S5 Fig Typical single-grain quartz luminescence results for sample SB153: a) Natural single-grain OSL signal, (b) a dose-response curve for the OSL signal measured on a single aliquot. Regenerated points are shown as full circles. The recycled point is shown as an open circle. The natural signal is shown as a triangle. (c) Single-grain OSL dose distribution displayed as a kernel density estimate (KDE) plot.
(TIF)

S6 Fig Abanico plot displaying single-grain quartz De distribution for SB153. The grey bar represents De values within 2 sigma. The three lines highlight different components of the three-component finite mixture model (FMM) fit, corresponding to distinct populations of grains within the broader De distribution. FMM 1 (pink) represents the most recent deposition of sediment for the sampled layer, FMM 2 (blue) corresponds to an intermediate dose population, and FMM 3 (green) represents a smaller and older grain population.
(TIF)

S7 Fig Comparison of K-rich feldspar pIR-IR_225_ ages (red open square), K-rich feldspar pIR-IR_290_ (red filled square) ages, multi-grain quartz OSL (dark blue circle) ages, and single-grain quartz OSL ages (blue triangle) for the MAR-T4 samples. Sample SB153 is the uppermost sample in the sequence, and SB155 is the lower most sample. Associated uncertainties are displayed at one sigma.
(TIF)

S8 Fig XRD results with identified minerals from samples a) MAR-T4.17 (columns) b) MAR-T4.18 (Columns) c) MAR-T4.19 d) MAR-T4.20 e) MAR-T4.21 f) MAR-T4.22 g) MAR-T4.23 h) MAR-T4.24. (TIF)

S9 Fig Micromorphological thin sections of A) MAR-T4.M3 B) MAR-T4.M1 C) MAR-T4.M4. (TIF)
