# Supplementary material for: A high-resolution, multi-method palaeoenvironmental record from pan sediments at Maralaleng Pan, southern Kalahari basin
Source: PLoS One. 2026 Sep 17;21(9):e0357461. doi: 10.1371/journal.pone.0357461 (PMC13585237; doi:10.1371/journal.pone.0357461)
Supplement: Supplementary file 1 [file pone.0357461.s001.docx]

Supplementary online material 1: Faul et al.,

**A high-resolution, multi-method palaeoenvironmental record from pan sediments at Maralaleng Pan, southern Kalahari basin.**

Inèz Faul^1^, Taylor Grandfield^2^, Chris Green^1^, Elena Hensel-Karamalakidou^3^, Phillip Segadika^4^, Rowena Winterhalder^1^, Sarah Mothulatshipi^5^, Stefan Dreibrodt^6,7^, Nils Andersen^8^, Marine Frouin^2^, Michaela Ecker^1,9^.

1 - Institute of Prehistoric and Protohistoric Archaeology, Kiel University, Kiel, Germany

2 - Department of Geosciences, Stony Brook University, USA

3 – Institute for Ecosystem Research, Kiel University, Kiel, Germany

4 - Department of National Museum and Monuments, Gaborone, Botswana

5 - Department of History, University of Botswana, Gaborone, Botswana

6 - State Office for Monument Preservation Baden-Württemberg, Germany

7 – Department of Geosciences, University of Tübingen, Germany

8 - Leibniz Laboratory for Radiometric Dating and Stable Isotope Research, Kiel University, Kiel, Germany

9 - Honorary Research Associate, Archaeology Department, McGregor Museum, Kimberley, South Africa

Content:

Supplementary Text:

Section S1: Biomarkers extended methods

Section S2: Luminescence dating, extended methods and discussion

Supplementary Tables S1 to S5

Supplementary Fig. S1 to S9

An additional Excel file with raw data from TIC/TOC, Magnetic susceptibility, XRD, ED-XRF, carbonate stable isotope data, plant wax biomarker data and the GRADISTAT data is available as a supplementary file (S3).

**Supplementary Text**

*S1 - Biomarkers extended methods*

The following GC-FID temperature program was used for samples with uneven numbers: the oven was maintained for 1 min at an initial temperature of 50°C, then the temperature was increased to 300°C (10 min hold) before a further increase to 330°C (10 min hold). For even numbered samples, the GC-FID temperature program was slightly adjusted; the oven was maintained for 1 min at an initial temperature of 50°C, then the temperature was increased to 150°C (5 min hold) before a further increase to 330°C (5 min hold).

The number of *n*-alkanes per sample shows a wide range across samples. However, the concentrations should not be taken as exact values and are an overestimation for samples with uneven numbered sample names. When measuring these stable isotopes in the mass spectrometer, we noticed that concentrations were by factor two lower across samples when compared to the results calculated from the gas chromatograph (GC) measurement. However, as this is a consistent overestimation that does not vary, the general trend in distribution of individual homologue abundances and indices calculations should not be affected. The even numbered samples were prepared with a new ASE machine and measured a year later (with a new internal standard) and there is no discrepancy in concentrations between GC and mass spectrometer measurements.

The carbon preference index (CPI) which has been calculated using the following formula:

CPI_25-35_ = [(C_25_+C_27_+C_29_+C_31_+C_33_) + (C_27_+C_29_+C_31_+C_33_+C_35_)] / [2*(C_26_+C_28_+C_30_+C_32_+C_34_)].

Average chain length (ACL) of the terrestrial plant input (which is mainly C_27_-C_33_ homologues) was calculated as:

ACL_27-33_ = (27 × C_27_ + 28 × C_28_ + 29 × C_29_ + 30 × C_30_ + 31 × C_31_ + 32 × C_32_ + 33 × C_33_) / (C_27_ + C_28_ + C_29_ + C_30_ + C_31_ + C_32_ + C_33_).

*Norm*31 is calculated as: *Norm*31=C_31_ / (C_29_ + C_31_) and *Norm*33 as: *Norm*33=C_33_ / (C_29_ + C_33_).

*S2 - Luminescence Dating*

In 2023, three sediment samples were collected at the site of Maralaleng (MAR) Pan in MAR-T4. The sampling was carried out under an artificial shelter using filtered red light. After thoroughly cleaning the first 2 cm of the exposed section, the luminescence samples were collected in opaque plastic bags using a trowel. Additional bulk sediment samples were collected next to each sampling location for dose rate determination. The samples were assigned unique identification numbers at the Luminescence Dating Research Laboratory, Stony Brook University (SB153, SB155, and SB156 in Fig. S1)

1. *Dosimetry*

For the external dose rate (Dr), the beta and gamma dose contributions were derived from the concentration of potassium (K), thorium (Th), and uranium (U), measured with a low-background, high resolution gamma spectrometer with a high-purity germanium detector (Mirion BEGe 3830), enclosed in a 6-inch-thick lead shield, and cooled with an electric cryostat (Cryo-Pulse 5 PLUS). Prior to measurement, the samples were oven-dried for 24 hours, then pulverized and homogenized using a shatterbox mill. Approximately 12 g of material was packed into standard radon (Rn)-tight plastic containers and sealed with epoxy to prevent ^222^Rn escape. To allow for secular equilibrium between ^222^Rn and its parent radium-226 (^226^Ra), the sealed containers were stored for at least 28 days before counting. The sealed samples were placed directly on the detector, centered for optimal alignment, and the counting duration for the samples was optimised to maximize the signal-to-noise ratio (between 7 to 10 days). Radioelement concentrations were derived from isotope peaks in the gamma spectrum and calibrated against certified IAEA and in-house standards (Table S1).

A key advantage of high-resolution gamma spectrometry, over bulk geochemical methods, is that it allows a direct assessment of secular equilibrium in the uranium decay series. Recent U uptake, Ra loss, or Radon escape can be identified by comparing activities or concentrations derived from the head of the chain and the tail of the chain. To diagnose the U-series disequilibrium, we first compared the concentration ratios U(^238^U)/^232^Th and U(^226^Ra)/^232^Th. Th is immobile under surficial conditions and therefore provides a stable baseline [1]. Fig. D2A reveals a marked difference between the basal and upper units. The lowermost sample (SB155) shows U(^238^U)/^232^Th and U(^226^Ra)/^232^Th ratios close to the 1:1 line (i.e. equilibrium), while the uppermost samples (SB156 and 153) display elevated U(^238^U)/^232^Th relative to U(^226^Ra)/^232^Th. This disequilibrium pattern is characteristic of secondary uranium enrichment [1], where the head of the chain is raised by U uptake into near-surface calcretes and organic matter. We can also take the U(^210^Pb)/U(^226^Ra) ratio into consideration to evaluate possible radon loss within the tail of the ^238^U decay series. Due to the fact that ^226^Ra decays to ^222^Rn, a noble gas with high mobility, any escape of radon from the sediment would deplete its short-lived daughters. In such cases, the activity of U(^210^Pb) falls below that of U(^226^Ra), and the dose rate contribution from the uranium chain is reduced. Ratios close to unity therefore indicate that radon and its daughters are retained and that the tail of the chain is in secular equilibrium. Values <1 reflect a partial radon loss and values >1 may indicate the addition of ^210^ Pb from atmospheric fallout. In MAR-T4, all ratios fall within the 1 ± 5%, (Fig. S2B), confirming that the post-radon portion of the chain is in equilibrium with ^226^Ra. In other words, the short-lived daughters have re-established balance with radium, and no ongoing radon loss is detectable.

The consequences for the dose rate estimation are as follows: The long-lived isotopes at the head of the chain (^238^U, ^234^U, ^230^Th), decay almost exclusively by α-emission, which contributes mainly to the internal α dose in mineral grains. By contrast, the tail of the chain produces the bulk of the β-particles and γ-rays that account for the external environmental dose. Dose-rate conversion tables show that roughly 80 - 90% of the uranium-series β/γ dose derives from these post-radon daughters [2], whereas the head contributes little beyond α. This distribution means that even if the head of the chain is enriched in uranium relative to ^226^Ra (as seen in MAR-T4, Fig. S2A), the disequilibrium has little effect on the β/γ dose delivered to the grains. In addition, since the post-radon daughters are in equilibrium with ^226^Ra (Fig. S2B), the β/γ contribution is accurately represented by bottom-chain activity. Therefore, for the dose rate calculation, we followed standard practice [3; 4; 5], assuming that ~90% of the U-series gamma and beta dose comes from the bottom-chain daughters.

The measurement of the water content ranged from 10 % to 30 % by weight at the time of sample collection, during the dry season in June 2023. To account for seasonal and long-term fluctuations, we adopted an average moisture content of 30 ± 10%. This 1 sigma range (20-40%) captures the variability expected under typical dry-wet season cycles, while the corresponding 2 sigma range (10-50%) encompasses the hydrological extremes, from peak pan saturation to duricrust formation. We consider 30 ± 10% to provide a geologically reasonable estimate of past moisture conditions relevant to dose rate attenuation.

Cosmic dose rate was determined based on the site’s location (altitude and latitude) and the burial depth of the sediments, after [6]. For the feldspar grains, the internal dose rate was estimated assuming a potassium and rubidium content of 12 ± 0.5% and 400 ± 100 ppm, respectively, and considering the alpha and beta grain size attenuation factors of [7] and [8], respectively. For quartz an internal dose rate of 0.03 ± 0.02 Gy.ka^−1^ was used. For each sample, the annual dose rate was calculated using the conversion factors after [2] in DRAC v1.2 [9].
The annual dose rates ranged from 2.1 ± 0.1 (SB153 and SB155) to 2.8 ± 0.2 [Gy.ka](http://gy.ka)^-1^ (SB156) for quartz, and 3.0 ± 0.2 (SB153) to 3.8 ± 0.2 [Gy.ka](http://gy.ka)^-1^ (SB156) for K-rich feldspar.

2. *Luminescence analysis strategy*

Multiple studies have emphasized the importance of comparing luminescence ages obtained from different signals to identify incomplete bleaching or anomalous fading, and to assess the reliability of final age estimates [10; 11; 12; 13]. At MAR-T4, the stratigraphy is complex, with the development of an incipient calcrete near the surface suggesting potential post-depositional disturbances, grain movement, and/or mixing. To address these challenges, we applied a multi-protocol strategy designed to track and interpret these effects. As [3;4] emphasized, luminescence dating requires flexibility in protocol choice, with different measurements applied as dictated by the material, context, and signal behaviour. In this study, we adopted a stepwise strategy, applying additional measurement protocols where initial results indicated potential problems such as incomplete bleaching, anomalous fading, or grain mixing.

Our investigation began with K-rich feldspar using two post-infrared IRSL protocols, stimulated at 225 °C (pIR-IR_225_; [14]) and 290 °C (pIR-IR_290_; [15]) to measure the equivalent dose (De). These provided an initial assessment of the suitability of feldspar signals dating, and also a way to compare signals with different bleaching sensitivities. Since feldspar is known to bleach slower than quartz when exposed to sunlight [16], ages derived from feldspar alone could overestimate the timing of deposition if signals were not fully reset. For this reason, we complemented feldspar analyses with quartz OSL measurements at both the multi-grain and single-grain level; the latter being especially effective for detecting partially bleached or mixed populations of grains. The combination of these approaches allow us to evaluate the extent to which bleaching and mixing influenced the De distributions and to cross-check the reliability of resulting age estimates. This adaptive, multi-protocol strategy ensured that potential complications introduced by the complex MAR-T4 stratigraphy were explicitly considered, increasing confidence in the final chronological interpretation. A summary of the De measurement protocols applied to each sample is presented in Table S2.

3. *Sample preparation*

The light-sensitive sediment samples were prepared for luminescence analysis in the laboratory darkroom under controlled low-intensity orange light conditions [17]. After an ultrasonic bath (duration of 10 min), the samples were wet sieved to isolate the 185-255 μm size fraction. The fraction was then chemically treated with hydrochloric acid (10% HCl) and hydrogen peroxide (30% H_2_O_2_) for 72 hr to remove carbonates and organic matter, respectively. Mineral separation was performed using lithium heteropolytungstate (LST) heavy liquid at successive densities: 2.58 g cm^-3^ to float K-rich feldspars, 2.62 g cm^-3^ for further plagioclase removal, and 2.75 g cm^-3^ to separate quartz from denser heavy minerals. Both quartz and K-rich feldspars were recovered from all samples. The quartz grains were subsequently treated with hydrofluoric acid (48% HF) for 40 min to remove any remaining feldspar and the α-irradiated outer rind, rinsed in 10% HCl to dissolve fluoride precipitates, dried, and re-sieved to 185 μm.

The purified quartz and K-feldspar fractions were used for luminescence analysis. For multi-grain De measurements, the grains were mounted on stainless steel cups, or discs, with a silicone oil adhesive of ~ 2 mm diameter. For single-grain analysis the grains were mounted into aluminium single-grain discs, where each disc containing 100 holes (300 µm in diameter and depth) drilled into a 10 x 10 grid with 600 µm spacing between the center of each hole. Each disc was inspected under a microscope before measurement to confirm single-grain loading.

4. *Felspar multi-grain*

Luminescence measurements were conducted using an automated OSL/TL Risø-DA-20 reader [18], aka ‘Mothership’, equipped with a photomultiplier tube (ET PDM9107-CP-TTL) and a ^90^Sr/^90^Y beta source delivering a dose rate of ca. 0.087 ± 0.002 Gy s^-1^ to the material deposited on the cups at the time of measurement (Risø calibration quartz batch 204). Infrared light-emitting diodes, emitting at 850 ± 30 nm, were employed to stimulate the K-rich feldspar fraction and the resulting luminescence signal was detected through a “blue filter pack” composed of a 2 mm thick Schott BG3 and a 2 mm thick Schott BG39 filter (detection window centred on ca. 410 nm) positioned in front of the photomultiplier tube. All measurements were performed under a nitrogen atmosphere.

The pIR-IR single-aliquot regenerative-dose (SAR) protocols were applied using four regenerative doses and a zero-dose cycle (recuperation) to build the dose-response curve, with the first regenerative dose point being repeated at the end to check for sensitivity changes (recycling ratio) after repeated measurement [19]. The net intensity of the IRSL was integrated over the first 2.5 s after subtracting the background signal derived from the last 10 s of stimulation. The De’s and their uncertainties were obtained using the Analyst software v. 4.57 [20] by interpolating the normalized natural OSL signal onto the normalized regeneration curve, fitted with a single exponential function.

For all the samples, the pIR-IR_225_ protocol was measured on eleven to twelve aliquots. For all aliquots, the recycling ratios were within 10% of unity and the recuperation was <5%, confirming the suitability of the SAR protocol for these samples. The over-dispersion (OD) values were determined using the Central Age Model (CAM) using the function cal_CentralDose() v.1.5 from the R ‘Luminescence’ package v.1.1.0 [21; 22; 23]. The OD quantifies the additional spread in De values that cannot be explained by analytical uncertainties alone and is reported as a percentage of the mean. For MAR-T4 samples, the lowermost sample (SB155) gave an OD of 9 ± 4 %, while the subsequent sample (SB156) gave an OD of 34 ± 8%, and the uppermost sample (SB153) gave the highest OD at 46 ± 11% (SB153) (Table S3).
In multi-grain aliquots, the luminescence signal reflects the combined response of many grains, typically less than one hundred grains in a 2 mm diameter stamp. Due to this averaging effect, and because feldspar grains are typically very bright, their strong signals tend to dominate the aliquot response, and mask differences between grains. As a result, OD values below ~10% for K-feldspar are generally interpreted as evidence of well-bleached, homogeneous samples [24]. The high OD values observed here for two samples require further consideration. First, they suggest that only a minority of feldspar grains emitted a measurable signal, causing the aliquot signal to be disproportionately influenced by a few grains and amplifying the apparent spread in De values. Second, sediment mixing after deposition is a plausible contributing factor, whether through bioturbation, pedogenic processes, or reworking of older material [25]. By contrast, microdosimetric heterogeneity, which can sometimes drive high OD through small-scale differences in dose rate, is less likely here because feldspar grains are bright enough that such effects typically average out at the multi-grain scale [25].

For the lowermost sample in the sequence, we decided to measure the pIR-IR_290_ signal to assess the bleaching level of grains. Twelve aliquots were measured, showing all recycling ratios within 10% of unity and recuperation values <5%. Examples of the pIR-IR decay, dose-response curves, and dose distributions for the pIR-IR_225_ and pIR-IR_290_ signals for sample SB155 are shown in Fig. S3. For the pIR-IR_290_, the OD value for SB155 was 11 ± 4%. For this sample, the two De’s values obtained 311.2 ± 11.4 Gy (pIR-IR_225_), and 351.3 ± 14.7 Gy (pIR-IR_290_) are very similar, with overlapping uncertainties - suggesting that these two signals have been sufficiently bleached.

5. *Quartz OSL multi-grain*

Luminescence measurements were conducted using an automated OSL/TL Risø-DA-20 reader [18] aka ‘Mothership’, equipped with a photomultiplier tube (ET PDM9107-CP-TTL) and a ^90^SR/^90^Y beta source delivering a dose rate of ca. 0.095 ± 0.003 Gy s^-1^ to the material deposited on the discs at the time of measurement (Risø calibration quartz batch 204). Blue light-emitting diodes (LEDs), emitting at 470 ± 30 nm, were employed to stimulate the quartz fraction and the resulting luminescence signal was detected through a combination of 2.5 and 5mm thick Hoya U-340 glass filters (transmission between ~290-370 nm) positioned in front of the photomultiplier tube. All measurements were performed under a nitrogen atmosphere.

The samples were measured using a standard multi-grain, SAR protocol [19; 26; 27]. For the quartz, dose recovery tests at different preheat temperatures were performed to determine the optimal protocol parameters for the De measurements. The preheat combination that gives the optimal results (given dose/measure dose within 10% of unity, low recuperation and recycling ratio within 10% of unity) was a preheat temperature of 220°C and a cut-heat temperature of 180°C. The protocol was applied using four regenerative doses and a zero-dose cycle (recuperation) to build the dose-response curve, with the first regenerative dose point being repeated at the end to check for sensitivity changes (recycling ratio) after repeated measurement [19]. An additional OSL IR depletion step was added to the end of each measurement to check for feldspar contamination [28].

The quartz OSL signal was measured for 40 s at 125°C. The net intensity of the blue luminescence signal was integrated over the first 0.8 s after subtracting the background signal derived from the subsequent 3.2 s of stimulation [29]. The De’s and their uncertainties were obtained using the Analyst software v. 4.57 [28] by interpolating the normalized natural OSL signal onto the normalized regeneration curve, fitted with a single exponential function.

The lower sample (SB155), only three aliquots were measured, and the natural luminescence signal (Ln/Tn) laid beyond the saturation level without intersecting the dose-response curve, and therefore an age determination for this sample was not possible. For SB153 and SB156, the OSL signal was measured on twelve aliquots per sample. For SB153 and SB156, the recycling ratios and IR depletion ratios were within 10% of unity and recuperation was <5% for all aliquots. An example of the multi-grain OSL signal, dose-response curve, and dose distribution for SB153 are displayed below in Fig. S4.

For SB156, the OD value is 18 ± 5%, which in OSL quartz multigrain is typically taken as evidence that the sample was well-bleached at deposition and has not been significantly affected by mixing [30]. In contrast, SB153 shows an OD of 57 ± 12%. Such elevated over dispersion value suggests substantial scatter in the De’s value and therefore required further assessment at the single grain level.

6. *Quartz OSL single-grain*

Subsequent analyses focused on SB153 to further evaluate the extent of mixing and/or incomplete bleaching. Single-grains of quartz were measured using an automated OSL/TL Risø-DA-20 reader, aka ‘Tardis’, equipped with a single-grain attachment [18; 31]. A green laser, emitting at 532 nm, was employed to stimulate each quartz grain and the resulting luminescence signal was detected through a combination of 2.5 and 5mm thick Hoya U-340 glass filters (transmission between ~290-370 nm) positioned in front of the photomultiplier tube. All measurements were performed under a nitrogen atmosphere. Spatial heterogeneity in dose delivery across individual grain positions was corrected following the guidance of [32].

The single-grain OSL signal was recorded for a total of 2 s, the OSL signal was integrated over the first 0.06 s of stimulation and the background was calculated from the subsequent 0.55s of stimulation. The De’s and their uncertainties were estimated using the Analyst software v. 4.57 [20] by interpolating the normalised natural OSL signal onto the normalised regeneration curve, fitted with a single exponential function.

The choice of single-grain OSL rejection criteria was guided by the need to balance data quality with retention of an adequate number of grains, following the recommendation of [33]. Standard rejection criteria, such as strict thresholds on recycling ratios, recuperation, and test-dose uncertainties, are widely applied in single-grain studies, but several authors have noted that their use can drastically reduce the number of accepted grains without necessarily improving the accuracy of the dose estimate. To establish criteria appropriate for our dataset, we first evaluated performance through dose recovery experiments. Based on these tests, we adopted the following thresholds on single-grain De values: (i) relative uncertainty on the first test dose signal < 20% [33], (ii) IR depletion ratio within 10% of unity [28] to screen for feldspar contamination, and (iii) De error <30% of the De value. Additional checks, such as recycling ratios and recuperation values, were also applied to reject grains; however, their inclusion had no measurable effect on the overall dose recovery performance, which remains 0.97 ± 0.02. This result supports the conclusion of [33] that rejection criteria should be tailored to the data rather than applied rigidly, as overly strict filtering may exclude grains that contribute valid information. Based on these results, we consider the criteria listed above to be the most appropriate for sample SB153. Of the 354 grains measured with a detectable OSL signal, 125 passed the rejection criteria (Table S5). An example of the single-grain OSL signal, dose-response curve, and dose distribution is displayed below in Fig. S5.

The single-grain De distribution (Fig. S6) shows a large scatter (OD > 100%) and clear multimodality. Such patterns are typical of samples affected by partial bleaching, mixing of population and/or heterogeneous microdosimetry [30;34]. We therefore explored a range of statistical models, including the CAM, the Average Dose Model (ADM, [35]), and the Minimum Age Model (MAM; [25]), but none isolated the principal mode satisfactorily. Given the clear multimodality, we applied a finite mixture model (FMM, [36], implemented with calc_FiniteMixture() v.0.4.3 from the R ‘Luminescence’ package v.1.1.0 [37;36;23]. As emphasised in recent community guidelines [38], the FMM should be used only when there is genuine evidence for discrete sub-populations and sufficient grain counts (n ≳ 50) to stabilise fits - criteria that are satisfied here. The FMM identified three components: 58 % of the grains cluster ~4 Gy (FMM1), 20% around ~11 Gy (FMM2), and 22 % around ~43 Gy (FMM3) (Figs. S5c, S6 and Table 4).

**Fig. S1**. OSL sampling locations for MAR-T4

**Fig. S2**. Assessment of the secular equilibrium in the uranium decay series of sediment samples SB153 (pink), SB156 (blue), SB155 (green), with a comparison between the (A) radioelement concentration derived from the head of the chain and the tail of the chain, and (B) radioelement concentration from the tail of the chain. The 1:1 equilibrium line is indicated by a black line, with dashed lines indicating ± 5%.

**Fig. S3**, Typical feldspar luminescence results for sample SB155: a) Natural pIR-IR_225_ signal, (b) a dose-response curve for the pIR-IR_225_ signal measured on a single aliquot. Regenerated points are shown as full circles. The recycled point is shown as an open circle. The natural signal is shown as a triangle. (c) pIR-IR_225_ dose distribution displayed as a kernel density estimate (KDE) plot. (d) Natural pIR-IR_290_ signal; (e) a dose-response curve for the pIR-IR_290_ signal measured on a single aliquot; (f) pIR-IR_290_ dose distribution displayed as a KDE plot.

**Fig. S4**. Typical multi-grain quartz luminescence results for sample SB153: a) Natural OSL signal, (b) a dose-response curve for the OSL signal measured on a single aliquot. Regenerated points are shown as full circles. The recycled point is shown as an open circle. The natural signal is shown as a triangle. (c) OSL dose distribution displayed as a kernel density estimate (KDE) plot.

**Fig. S5**. Typical single-grain quartz luminescence results for sample SB153: a) Natural single-grain OSL signal, (b) a dose-response curve for the OSL signal measured on a single aliquot. Regenerated points are shown as full circles. The recycled point is shown as an open circle. The natural signal is shown as a triangle. (c) Single-grain OSL dose distribution displayed as a kernel density estimate (KDE) plot.

**Fig. S6.** Abanico plot displaying single-grain quartz De distribution for SB153. The grey bar represents De values within 2 sigma. The three lines highlight different components of the three-component finite mixture model (FMM) fit, corresponding to distinct populations of grains within the broader De distribution. FMM 1 (pink) represents the most recent deposition of sediment for the sampled layer, FMM 2 (blue) corresponds to an intermediate dose population, and FMM 3 (green) represents a smaller and older grain population.

**Fig. S7.** Comparison of K-rich feldspar pIR-IR_225_ ages (red open square), K-rich feldspar pIR-IR_290_ (red filled square) ages, multi-grain quartz OSL (dark blue circle) ages, and single-grain quartz OSL ages (blue triangle) for the MAR-T4 samples. Sample SB153 is the uppermost sample in the sequence, and SB155 is the lower most sample. Associated uncertainties are displayed at one sigma.

**Table S1:** Environmental dose rate information for sediment samples at MAR-T4.

| **SB#** | **Depth**  **(m)** | **Radioelement Concentration** | | | **Dose rate (Gy.ka^-1^)^b^** | | | | |
| --- | --- | --- | --- | --- | --- | --- | --- | --- | --- |
|  |  | **K**  **(%)** | **U**  **(eq.ppm)** | **Th**  **(eq.ppm)** | **Ext.**  **Beta** | **Gamma** | **Cosmic** | **Total for quartz ^c^** | **Total for KF ^c^** |
| SB153 | 0.42 | 0.57 ± 0.01 | 7.29 ± 0.22 | 1.39 ± 0.06 | 1.00 ± 0.09 | 0.77 ± 0.07 | 0.24 ± 0.02 | 2.1 ± 0.1 | 3.0 ± 0.2 |
| SB156 | 0.87 | 0.97 ± 0.02 | 9.88 ± 0.13 | 1.74 ± 0.11 | 1.45 ± 0.13 | 1.07 ± 0.09 | 0.21 ± 0.02 | 2.8 ± 0.2 | 3.8 ± 0.2 |
| SB155 | 1.45 | 1.04 ± 0.02 | 6.29 ± 0.26 | 1.95 ± 0.08 | 1.13 ± 0.12 | 0.78 ± 0.07 | 0.19 ± 0.02 | 2.1 ± 0.1 | 3.1 ± 0.2 |

^a^ K, U, and Th content have been determined by gamma spectrometry.

^b^ Dose rates were calculated using DRAC v.1.2 [9] with the following parameters: conversion factors from [2] and beta- dose attenuation from [8], and external dose rates include adjustments for the water content (30 ± 10%).

^C^ The internal dose rate for K-rich feldspar is 0.90 ± 0.14 [Gy.ka](http://gy.ka)^-1^, 0.03 ± 0.02 [Gy.ka](http://gy.ka)^-1^ for quartz

**Table S2**. Overview equivalent dose measurement protocols applied to each sample,

| **SB#** | **Depth (m)** | **Quartz** | | **K-rich Feldspar** | |
| --- | --- | --- | --- | --- | --- |
|  |  | **Multi-grain OSL** | **Single-grain OSL** | **Multi-grain pIR-IR_225_** | **Multi-grain pIR-IR_290_** |
| SB153 | 0.42 | X | X | X |  |
| SB156 | 0.87 | X |  | X |  |
| SB155 | 1.45 | X |  | X | X |

**Table S3**. Summary of equivalent doses and ages of K-rich feldspars at MAR-T4.

| **SB#** | **Depth**  **(m)** | **pIR-IR_225_** | | | | **pIR-IR_290_** | | | |
| --- | --- | --- | --- | --- | --- | --- | --- | --- | --- |
|  |  | **n^a^** | **De^b^**  **(Gy)** | **OD^b^**  **(%)** | **Age^c^**  **(ka)** | **n^a^** | **De^b^**  **(Gy)** | **OD^b^**  **(%)** | **Age^c^**  **(ka)** |
| SB153 | 0.42 | 11(11) | 27.1 ± 3.9 | 46 ± 11 | 9.0 ± 1.4 | - | - | - | - |
| SB156 | 0.87 | 12(12) | 116.1 ± 12.5 | 34 ± 8 | 31.0 ± 3.8 | - | - | - | - |
| SB155 | 1.45 | 12(12) | 311.2 ± 11.4 | 9 ± 4 | 101.0 ± 7.3 | 12(12) | 351.3 ± 14.7 | 11 ± 4 | 114.1 ± 8.5 |

^a^ Number of aliquots that passed the selection criteria and used for the calculation of the De. The total number of aliquots measured is under parentheses.

^b^ The final equivalent dose (De) and overdispersion (OD) values were calculated using the Central Age Model (CAM, [25]) using the function calc_CentralDose() v.1.5 from the R ‘Luminescence’ package v.1.0.1 [23;37]. Uncertainties are given at one sigma.

^c^ Final age results were calculated using DRAC v.1.2 [9]

**Table S4**. Summary of equivalent doses and ages for Quartz at MAR-T4.

| **SB#** | **Quartz Multi-grain** | | | | **Quartz Single-grain** | | | |
| --- | --- | --- | --- | --- | --- | --- | --- | --- |
|  | **n^a^** | **De^b^**  **(Gy)** | **OD^b^**  **(%)** | **Age^c^**  **(ka)** | **n^d^** | **Proportion^e^**  **(%)** | **De^e^**  **(Gy)** | **Age^c^**  **(ka)** |
| SB153 | 12(11) | 7.2 ± 1.2 | 57 ± 12 | 3.5 ± 0.6 | 125(354) | 58 (FMM1)  20 (FMM2)  22 (FMM3) | 3.68 ± 0.1  11.12 ± 0.5  43.34 ± 1.49 | 1.8 ± 0.1  5.4 ± 0.4  21.1 ± 1.4 |
| SB156 | 12(12) | 90.2 ± 5.2 | 18 ± 5 | 32.4 ± 2.7 | - | - | - | - |
| SB155 | - (3) | Saturated | - | - | - | - | - | - |

^a^ Number of aliquots that passed the selection criteria and used for the calculation of the De. The total number of aliquots measured is under parentheses.

^b^ The final equivalent dose (De) and overdispersion (OD) values were calculated using the Central Age Model (CAM, Galbraith et al., 1999) using the function calc_CentralDose() v.1.5 from the R ‘Luminescence’ package v.1.0.1 [21; 23]. Uncertainties are given at one sigma.

^c^ Final age results were calculated using DRAC v.1.2 [9]

^d^ Number of grains that passed the selection criteria and used for the calculation of the De. The total number of grains measured is under parentheses.

^e^ The final equivalent dose (De) was calculated using the Finite Mixture Model (FMM, Galbraith et al., 1990) using a three component model using the function calc_FiniteMixture() v.04.3. from the R ‘Luminescence’ package v.1.0.1 [21; 23]. The resulting De is from the FMM component 1 that best represents the primary peak of the De distribution, uncertainty is given at one sigma.

**Table S5**. Single-grain quartz rejection criteria summary

| **Rejection Criteria - SB153** | |
| --- | --- |
| ***Summary:*** | |
| Total number of discs measured | *18* |
| Total number of grains measured | *354* |
| Total number of accepted grains | *125* |
| Acceptance rate (%) | *35* |
| ***Number of grains failing each individual criterion ^a^*** | |
| S_TN_<20% | *157* |
| IR Depletion Ratio (1.0 ± 0.1) | *43* |
| De Error > 30% of De Value | *213* |

^a^An individual grain may fail multiple criterion, but is only accepted if it passes all three

Fig. S8. XRD results with identified minerals from samples A) MAR-T4.17 (columns) B) MAR-T4.18 (Columns) C) MAR-T4.19 D) MAR-T4.20 E) MAR-T4.21 F) MAR-T4.22 G) MAR-T4.23 H) MAR-T4.24.

Fig. S9. Micromorphological thin sections of A) MAR-T4.M3 B) MAR-T4.M1 C) MAR-T4.M4.

**References:**

1. Guibert P, Lahaye C, Bechtel F. The importance of U-series disequilibrium of sediments in luminescence dating: A case study at the Roc de Marsal Cave (Dordogne, France). Radiat Meas. 2009; 44(3): 223–31.
2. Guérin G, Mercier N, Adamiec G. Dose-rate conversion factors: update. Ancient TL. 2011; 29(1): 5–8.
3. Aitken MJ. Thermoluminescence dating. U.S. ed. London: Academic Press; 1985.
4. Aitken MJ. Introduction to optical dating: the dating of Quaternary sediments by the use of photon-stimulated luminescence. Oxford: Clarendon Press; 1998.
5. Lahaye C. Nouveaux apports de la thermoluminescence à la chronologie du Paléolithique dans le Sud-Ouest de la FranceEtudes en milieu hétérogène et en présence de déséquilibres radioactifs dans les séries de l’uranium [Internet] [These de doctorat]. Bordeaux 3; 2005 [cited 2025 Sep 2]. Available from: <https://theses.fr/2005BOR30071>
6. Prescott JR, Hutton JT. Cosmic ray and gamma ray dosimetry for TL and ESR. Int J Radiat Appl Instrum Part D Nucl Tracks Radiat Meas. 1988; 14(1): 223–7.
7. Brennan BJ, Lyons RG, Phillips SW. Attenuation of alpha particle track dose for spherical grains. Int J Radiat Appl Instrum Part D Nucl Tracks Radiat Meas. 1991; 18(1): 249–53.
8. Guérin G, Mercier N, Nathan R, Adamiec G, Lefrais Y. On the use of the infinite matrix assumption and associated concepts: A critical review. Radiat Meas. 2012; 47(9): 778–85.
9. Durcan JA, King GE, Duller GAT. DRAC: Dose Rate and Age Calculator for trapped charge dating. Quat. Geochronol. 2015; 28: 54–61.
10. Frouin M, Lahaye C, Hernandez M, Mercier N, Guibert P, Brenet M, et al. Chronology of the Middle Palaeolithic open-air site of Combe Brune 2 (Dordogne, France): a multi luminescence dating approach. J Archaeol Sci. 2014; 52: 524–34.
11. Guérin G, Frouin M, Talamo S, Aldeias V, Bruxelles L, Chiotti L, et al. A multi-method luminescence dating of the Palaeolithic sequence of La Ferrassie based on new excavations adjacent to the La Ferrassie 1 and 2 skeletons. J Archaeol Sci. 2015; 58: 147–66.
12. Tribolo C, Mercier N, Dumottay C, Cantin N, Banks WE, Stratford D, et al. Luminescence dating at Border Cave: attempts, questions, and new results. Quat Sci Rev. 2022; 296: 107787.
13. Taratunina NA, Buylaert JP, Kurbanov RN, Yanina TA, Makeev AO, Lebedeva MP, et al. Late Quaternary evolution of lower reaches of the Volga River (Raygorod section) based on luminescence dating. Quat Geochronol. 2022; 72: 101369.
14. Buylaert JP, Murray AS, Thomsen KJ, Jain M. Testing the potential of an elevated temperature IRSL signal from K-feldspar. Radiat Meas. 2009; 44(5): 560–5.
15. Thiel C, Buylaert JP, Murray A, Terhorst B, Hofer I, Tsukamoto S, et al. Luminescence dating of the Stratzing loess profile (Austria) – Testing the potential of an elevated temperature post-IR IRSL protocol. Quat Int. 2011; 234(1): 23–31.
16. Murray A, Thomsen K, Masuda N, Buylaert JP, Jain M. Identifying well-bleached quartz using the different bleaching rates of quartz and feldspar luminescence signals. Radiat Meas. 2012; 47: 688–95.
17. Frouin M, Grandfield T, Huebsch W, Evans O. Technical note: Darkroom lighting for luminescence dating laboratory. Geochronology. 2023; 5(2): 405–12.
18. Bøetter-Jensen L, McKeever SWS, Wintle AG. Optically Stimulated Luminescence Dosimetry. Amsterdam: Elsevier; 2003.
19. Wintle AG, Murray A. A review of optically stimulated luminescence characteristics and their relevance in single-aliquot regeneration dating protocols. Radiat Meas. 2006; 41: 369–91.
20. Duller GAT. The Analyst software package for luminescence data: overview and recent improvements. Ancient TL. 2015; 33(1): 35–42.
21. Burow, C., 2025. calc_CentralDose(): Apply the central age model (CAM) after Galbraith et al. (1999) to a given De distribution. Function version 1.5. In: Kreutzer, S., Burow, C., Dietze, M., Fuchs, M.C., Schmidt, C., Fischer, M., Friedrich, J., Mercier, N., Philippe, A., Riedesel, S., Autzen, M., Mittelstrass, D., Gray, H.J., Galharret, J., Colombo, M., Steinbuch, L., Boer, A.d., 2025. Luminescence: Comprehensive Luminescence Dating Data Analysis. R package version 1.1.0. <https://r-lum.github.io/Luminescence/>
22. Galbraith RF, Laslett GM. Statistical models for mixed fission track ages. Radiat Meas. 1993; 21(4): 459-70.
23. Kreutzer S, Burow C, Dietze M, Fuchs M, Schmidt C, Fischer M, et al. Luminescence: Comprehensive Luminescence Dating Data Analysis. R package version 1.1.0. 2025. <https://r-lum.github.io/Luminescence/>.
24. Smedley RK, Buylaert JP, Újvári G. Comparing the accuracy and precision of luminescence ages for partially-bleached sediments using single grains of K-feldspar and quartz. Quat Geochronol. 2019; 53: 101007.
25. Galbraith RF, Roberts RG, Laslett GM, Yoshida H, Olley JM. Optical Dating of Single and Multiple Grains of Quartz from Jinmium Rock Shelter, Northern Australia: Part I, Experimental Design and Statistical Models*. Archaeometry. 1999;41(2):339–64.
26. Murray AS, Wintle AG. Luminescence dating of quartz using an improved single-aliquot regenerative-dose protocol. Radiat Meas. 2000; 32(1): 57–73.
27. Murray A, Wintle A. The single aliquot regenerative dose protocol: Potential for improvements in reliability. Radiat Meas. 2003 ;37: 377–81.
28. Duller GAT. Distinguishing quartz and feldspar in single grain luminescence measurements. Radiat Meas. 2003; 37(2): 161–5.
29. Cunningham AC, Wallinga J. Selection of integration time intervals for quartz OSL decay curves. Quat Geochronol. 2010; 5(6): 657–66.
30. Galbraith RF, Roberts RG, Yoshida H. Error variation in OSL palaeodose estimates from single aliquots of quartz: a factorial experiment. Radiat Meas. 2005; 39(3): 289–307.
31. Duller GAT, Bøtter-Jensen L, Kohsiek P, Murray AS. A High-Sensitivity Optically Stimulated Luminescence Scanning System for Measurement of Single Sand-Sized Grains. Radiat Prot Dosimetry. 1999; 84(1–4): 325–30.
32. Ballarini M, Wintle AG, Wallinga J. Spatial variation of dose rate from beta sources as measured using single grains. Ancient TL. 2006; 24(1): 1–7.
33. Thomsen KJ, Murray AS, Buylaert JP, Jain M, Hansen JH, Aubry T. Testing single-grain quartz OSL methods using sediment samples with independent age control from the Bordes-Fitte rockshelter (Roches d’Abilly site, Central France). Quat Geochronol. 2016; 31: 77–96.
34. Duller GAT. Single-grain optical dating of Quaternary sediments: Why aliquot size matters in luminescence dating. Boreas. 2008; 37: 589–612.
35. Guérin G, Christophe C, Philippe A, Murray AS, Thomsen KJ, Tribolo C, et al. Absorbed dose, equivalent dose, measured dose rates, and implications for OSL age estimates: Introducing the Average Dose Model. Quat Geochronol. 2017; 41: 163–73.
36. Galbraith RF, Green PF. Estimating the component ages in a finite mixture. Int J Radiat Appl Instrum D. 1990; 17(3): 197–206.
37. Burow, C., Colombo, M., 2025. calc_FiniteMixture(): Apply the finite mixture model (FMM) after Galbraith (2005) to a given De distribution. Function version 0.4.3. In: Kreutzer, S., Burow, C., Dietze, M., Fuchs, M.C., Schmidt, C., Fischer, M., Friedrich, J., Mercier, N., Philippe, A., Riedesel, S., Autzen, M., Mittelstrass, D., Gray, H.J., Galharret, J., Colombo, M., Steinbuch, L., Boer, A.d., 2025. Luminescence: Comprehensive Luminescence Dating Data Analysis. R package version 1.1.0. <https://r-lum.github.io/Luminescence/>
38. Mahan S, Rittenour T, Nelson M, Ataee N, Brown N, DeWitt R, et al. Guide for interpreting and reporting luminescence dating results. Geol Soc Am Bull. 2023; 135 (5-6): 1480-502.
